# Coenzyme A fueling with pantethine limits autoreactive T cell pathogenicity in experimental neuroinflammation

**DOI:** 10.1186/s12974-024-03270-w

**Published:** 2024-11-05

**Authors:** Stefano Angiari, Tommaso Carlucci, Simona L. Budui, Simone D. Bach, Silvia Dusi, Julia Walter, Elena Ellmeier, Alyssa Schnabl, Anika Stracke, Natalie Bordag, Cansu Tafrali, Rina Demjaha, Michael Khalil, Gabriele Angelini, Eleonora Terrabuio, Enrica C. Pietronigro, Elena Zenaro, Carlo Laudanna, Barbara Rossi, Gabriela Constantin

**Affiliations:** 1 Otto Loewi Research Center, Division of Immunology, Medical University of Graz, Neue Stiftingtalstraße 6, 8010 Graz, Austria; 2 Department of Medicine, Section of General Pathology, University of Verona, Strada le Grazie 8, 37134 Verona, Italy; 3 Department of Dermatology and Venereology, Medical University of Graz, Graz, Austria; 4 Department of Neurology, Medical University of Graz, Graz, Austria; 5 The Center for Biomedical Computing (CBMC), University of Verona, Verona, Italy

**Keywords:** CoA metabolism, Pantethine, Autoreactive T cells, EAE, Multiple sclerosis, Immunometabolism

## Abstract

**Background:**

Immune cell metabolism governs the outcome of immune responses and contributes to the development of autoimmunity by controlling lymphocyte pathogenic potential. In this study, we evaluated the metabolic profile of myelin-specific murine encephalitogenic T cells, to identify novel therapeutic targets for autoimmune neuroinflammation.

**Methods:**

We performed metabolomics analysis on actively-proliferating encephalitogenic T cells to study their overall metabolic profile in comparison to resting T cells. Metabolomics, phosphoproteomics, in vitro functional assays, and in vivo studies in experimental autoimmune encephalomyelitis (EAE), a mouse model of multiple sclerosis (MS), were then implemented to evaluate the effect of metabolic targeting on autoreactive T cell pathogenicity. Finally, we confirmed the translational potential of our targeting approach in human pro-inflammatory T helper cell subsets and in T cells from MS patients.

**Results:**

We found that autoreactive encephalitogenic T cells display an altered coenzyme A (CoA) synthesis pathway, compared to resting T cells. CoA fueling with the CoA precursor pantethine (PTTH) affected essential immune-related processes of myelin-specific T cells, such as cell proliferation, cytokine production, and cell adhesion, both in vitro and in vivo. Accordingly, pre-clinical treatment with PTTH before disease onset inhibited the development of EAE by limiting T cell pro-inflammatory potential in vivo. Importantly, PTTH also significantly ameliorated the disease course when administered after disease onset in a therapeutic setting. Finally, PTTH reduced pro-inflammatory cytokine production by human T helper 1 (Th1) and Th17 cells and by T cells from MS patients, confirming its translational potential.

**Conclusion:**

Our data demonstrate that CoA fueling with PTTH in pro-inflammatory and autoreactive T cells may represent a novel therapeutic approach for the treatment of autoimmune neuroinflammation.

**Supplementary Information:**

The online version contains supplementary material available at 10.1186/s12974-024-03270-w.

## Background

The immune system protects the human body from infectious diseases and tissue insults, avoiding excessive inflammatory reactions. When mechanisms controlling this system fail, immune cells attack the body’s own tissues, resulting in the onset of autoimmunity. Most autoimmune diseases develop because of a complex mix of genetic predisposition and environmental triggers, resulting in a breakdown of immune tolerance [45]. Accordingly, the principal hallmark of autoimmunity is the presence of autoreactive adaptive immune cells that cause a systemic or local inflammatory response promoting tissue damage [45].

Intracellular metabolic reprogramming in immune cells governs the outcome of immune responses [30, 33]. In particular, it has been recently elucidated how aerobic glycolysis and other metabolic pathways control the functionality of T lymphocytes [5, 19]. Resting T cells show low levels of glycolysis, amino acid metabolism and fatty acid oxidation, which fuel the tricarboxylic acid (TCA) cycle and subsequent oxidative phosphorylation to generate adenosine triphosphate (ATP) as main energy source essential for their survival and quiescence. However, T cells profoundly change their metabolic profile upon activation, shifting to an anabolic metabolism that supports their proliferation and engagement of effector functions [5, 19]. T cell metabolic remodeling is controlled by the activity of key signaling pathways, including those regulated by the kinases mammalian target of rapamycin (mTOR), phosphoinositide 3-kinase (PI3K), and 5’ AMP-activated protein kinase (AMPK) [5, 19, 51]. Noteworthy, targeting metabolic enzymes and nutrient transporters or receptors, as well as the aforementioned signaling pathways, is emerging as a novel and intriguing approach to limit T cell-mediated inflammation and autoimmunity by altering their metabolic activity [34, 36, 59].

Myelin-specific encephalitogenic T cells are major players in the development of multiple sclerosis (MS) and its main animal model, experimental autoimmune encephalomyelitis (EAE). During EAE and MS, encephalitogenic T cells migrate in the central nervous system (CNS), where they attack the myelin sheath wrapping around axons and cause neuronal damage and death, which in turn leads to disability [4, 26]. Previous work identified profound metabolic remodeling in T cells from both MS patients and EAE mice, and targeting such metabolic alterations was proposed as an innovative strategy to limit autoimmune neuroinflammation [7, 50]. In this study, we evaluated the metabolic profile of murine actively-proliferating, myelin-specific encephalitogenic T cells, and identified a previously unappreciated potential role for coenzyme A (CoA) metabolism in their metabolic remodeling and pathogenic activity. We observed that CoA synthesis pathway is altered in encephalitogenic T cells, and demonstrated that CoA fueling with pantethine (PTTH), a CoA precursor [10, 43, 60], remodeled their intracellular metabolic profile and caused a strong immunomodulatory effect that reduced their overall pathogenic potential. Accordingly, PTTH inhibited neuroinflammation in EAE mice by limiting T cell activation, pro-inflammatory cytokine production, and integrin-mediated adhesion to the inflamed CNS microvasculature. Importantly, PTTH also reduced pro-inflammatory cytokine production by human Th1 and Th17 cells, as well as by T cells from MS patients, highlighting its translational value. Our work thus supports the potential of CoA fueling as a new strategy to dampen autoreactive T cell pathogenicity and inhibit autoimmune neuroinflammation.

## Methods

### Mice

8–10 weeks old SJL and C57Bl/6 mice were purchased from Charles River Laboratories and used for all experiments. All animal experiments were supervised by the local Institutional Animal Care Committee (OPBA) of the University of Verona and were conducted following the principles of the European Union’s Directive 2010/63.

### PTTH preparation

D-pantethine (PTTH) was purchased from Sigma-Aldrich. For in vitro experiments, PTTH was dissolved in phosphate-buffered saline (PBS) to a final concentration of 100 mM and stored at − 20 °C before use. For in vivo experiments, PTTH was dissolved in physiologic saline solution (0.9% NaCl in water solution) to a final concentration of 300 mg/ml and stored at − 20 °C before use.

### Production of PLP_139-151_-specific T cell lines

SJL mice were immunized subcutaneously (s.c.) with 300 μg of proteolipid protein (PLP)_139–151_ peptide (Genscript, USA) in 200 μl emulsion consisting of equal volumes of PBS and complete Freund’s adjuvant (CFA, Difco Laboratories), supplemented with 1 mg/ml of *Mycobacterium tuberculosis* (strain H37Ra; Difco Laboratories). 10–12 days later, draining lymph nodes (LNs) were collected and total cells were cultured in the presence of 30 μg/ml of PLP_139-151_ peptide for 4 days in complete medium [RPMI 1640 (Corning) supplemented with 1 mM sodium pyruvate (Sigma Aldrich), 4 mM GlutaMAX-I^®^ supplement (Thermo Fisher Scientific), 100 U/ml penicillin/streptomycin (Sigma Aldrich), 10% v/v fetal bovine serum (FBS; Thermo Fisher Scientific) and 5 μg/ml plasmocin (InvivoGen)]. After 4 days, cells were collected, washed, and re-plated in fresh complete medium. PLP_139-151_-specific T cell lines were obtained by re-stimulation of these cultures every 14 days for at least 3 times in the presence of irradiated splenocytes as antigen presenting cells (APCs - ratio 1:8) and 30 μg/ml of PLP_139-151_ peptide. Cells collected 2 days post stimulation (dps) were considered as “actively-proliferating”, while those collected 10–12 dps were considered as “non-proliferating”.

### Metabolomics analysis

Resting total CD4^+^ T cells were enriched via negative selection from LNs and spleens of 33 SJL mice using the CD4^+^ T cell isolation kit, mouse (Miltenyi Biotech), following manufacture instructions, and divided in four batches. PLP_139-151_-specific T cells were obtained from four independent actively-proliferating encephalitogenic T cell lines. Resting CD4^+^ T cells and PLP_139-151_-specific T cells were collected, washed twice with PBS, and pelleted. Cell pellets were immediately frozen in liquid nitrogen and stored at − 80 °C. In some experiments, resting CD4^+^ T cells and actively-proliferating encephalitogenic T cells were washed with PBS, re-suspended in fresh medium and treated with PBS or PTTH 1.0 mM for 6 h or 12 h before freezing. Total CoA levels were measured using the CoA assay kit (Abcam), following manufacturer’s instructions. Metabolite identification and quantification in T cells were performed in outsourcing by Metabolon (http://www.metabolon.com/). Metabolomics data visualization and statistical analysis were performed with R v4.3.1 [42] (using the packages readxl, openxlsx, stringr, plyr, dplyr, tidyr, tibble, reshape2, car, missMDA, colorspace, RColorBrewer, ggplot2, ggpmisc, ggrepel, ggforce, grid, scales, mixOmics, memoise, igraph, metap, MetaboAnalystR 4.0.0, nlme, emmeans) and TIBCO Spotfire v14.0.0 (TIBCO, Palo Alto, CA). Metabolites with less than < 30% missing data in all groups were used for multivariate analysis and univariate analysis (MVA and UVA, 137 metabolites; Supplementary data 1). Metabolites with data not missing at random were used only for univariate analysis in specific groups (66 metabolites). Six metabolites with very low signal intensities or high percentage of missing data were excluded from analysis. Samples were normalized to the median intensity of all MVA metabolites (metabolites before scaled 0 to 1) within the sample to correct for cell number differences. Median normalized intensities were log_10_-transformed and all normalized, transformed metabolites were normally distributed (Kolmogorov Smirnov test *p* > 0.05) and homoscedastic (Brown-Forsythe Levene-type test *p* > 0.05). All samples were checked for analytical outlier behavior according to the sample median, protein content and position in principal component analysis (PCA) excluding the first sample (UNVE-00062) from further analysis.

PCA was performed centered and scaled using the inbuilt default missing imputation with mixOmics::pca [44]. For partial least-squares discriminant analysis (PLS-DA), missings were imputed with missMDA::imputePCA [21] and data was centered and scaled for mixOmics::plsda [44]. Significance of group differences was tested by receiver operating characteristic (ROC) with mixOmics::auroc [44] using the first two components delivering area under the curve (AUC) of each group against all other groups and *p*-values from a Wilcoxon test between the predicted scores.

For univariate analysis, three factors had to be considered: CELL {naïve, PLP}, TREATMENT {CTRL, PTTH} and incubation TIME {6 h, 12 h}. The analysis was performed in a way that same CELL and TREATMENT conditions are considered as one group, irrespective of incubation TIME since the metabolomes from different incubation TIME were highly similar to each other in contrast to the two other biological factors in both PCA and PLS-DA. To investigate and correct for the potential additional variability contributed by the incubation TIME, linear mixed models with the random factor ~ 1|TIME {6 h, 12 h} and the fixed factors CELL and TREATMENT, as well as their interaction, were fitted with nlme::lme. To avoid potential overfitting, additionally generalized least squares models for only the fixed factors CELL and TREATMNT without random factor were fitted as well with nlme::gls [40]. Model performance was compared within each metabolite for lower Akaike information criterion, higher log-likelihood (goodness of fit), significance in log likelihood ratio test and direct comparison of fitted values. The model CELL*TREATMENT ~ 1|TIME showed the best fit for most metabolites and results are reported throughout. Results of pairwise comparisons of interest were extracted with emmeans::emmeans and *p*-values were False Discovery Rate (FDR) multiple test adjusted. For UVA graded metabolites with specific missing group, accordingly simplified models were used (see Supplementary Data 1).

For quantitative enrichment analysis (QEA) with MetaboAnalystR [35], all included metabolites were manually matched to their respective CAS, PUBCHEM ID, HMDB ID, KEGG ID and HMDB conform compound names using said databases and the online MetaboAnalyst Compound ID Conversion tool. Missing data was imputed with ImputeMissingVar(…, method = “knn_var”), data was autoscaled with Normalization(…, “AutoNorm”) and all measured metabolites were set as specific background calculating the global test result against SMPDB and RaMP pathways with CalculateGlobalTestScore [20]. For pathway topological analysis against KEGG pathways, the global test without-degree centrality was calculated with CalculateQeaScore [20]. All pathway results can be found in Supplementary Data 1.

### Phosphoproteomics and network analysis

After 6 h of treatment with PBS or PTTH 1.0 mM, actively-proliferating PLP_139-151_-specific T cells were washed twice with PBS and pelleted. Pellets were immediately frozen in liquid nitrogen and stored at − 80 °C. Analysis of protein expression and phosphorylation levels were performed in duplicate in outsourcing by Kinexus (http://www.kinexus.ca), using a Kinex™ KAM-850 Antibody Microarray Kit. The Kinex™ Antibody Microarray Service monitored changes in the expression levels and phosphorylation states of signaling proteins with more than 850 antibodies, which includes approximately 517 pan-specific antibodies (for protein expression evaluation) and 337 phospho-site-specific antibodies (for phosphorylation states evaluation). For each antibody, the background-corrected raw intensity data were logarithmically transformed with base 2. Furthermore, Z scores were calculated by subtracting the overall average intensity of all spots within a sample from the raw intensity for each spot, and dividing it by the standard deviations (SD) of all of the measured intensities within each sample [13]. Z ratios were further calculated by taking the difference between the averages of the observed protein Z scores and dividing it by the SD of all of the differences for comparisons between actively-proliferating PLP_139-151_-specific T cell treated or not with PTTH. A Z ratio of ± 1.1 was inferred as significant and used for bioinformatics analysis.

Gene Onthology (GO) enrichment analysis was performed by using GOnet [41]. The analysis was done on significant up- or down-regulated proteins. Only terms with *p*-value < 0.001 and FDR < 0.05 were considered. A bioinformatic probe, only including the set of eight down-regulated proteins, was extrapolated from the previous analysis and used as input for the subsequent topological network analysis. Topological network analysis was performed by using Cytoscape network analysis package (https://cytoscape.org). A global human PPI interactome dataset consisting of 19,439 proteins and 766,811 undirected binary interactions was compiled by integrating four global PPI datasets (http://iid.ophid.utoronto.ca, https://thebiogrid.org; https://cbdm-01.zdv.uni-mainz.de/~mschaefer/hippie/; https://apid.dep.usal.es). The compiled dataset included only experimentally determined interactions. Protein and interaction duplicates, and self-loops were removed with Cytoscape core tools. All protein IDs were normalized to HGNC nomenclature (https://www.genenames.org). A first interrogation of the global human PPI interactome dataset with the set of eight down-regulated proteins led to a first neighbors (FN) network of 1764 signaling proteins and 82,221 binary interactions. A topological network analysis procedure was then applied to reduce the complexity of the FN interactome. Four topological indexes of node centrality (centroid, betweenness, bridge and degree) and heat diffusion were computed by using the Cytoscape app Centiscape. The selection of proteins having topological indexes equal to or higher than 50% of the average led to a subnetwork of 487 proteins and 1587 interactions. Further interrogation of this sub-network with the set of eight down-regulated proteins coupled to Signor edge direction enrichment performed by using the Cytoscape core tools, led to a restricted directed signal transduction sub-network consisting of 51 signaling proteins and 105 interactions. Final network modularization with MCODE led to the identification of a highly interconnected directed signaling module of 6 proteins (all mitogen-activated protein kinases—MAPKs) and 15 interactions. Reactome pathway enrichment was then performed on these two signaling networks.

### Proliferation assays

1. In vitro proliferation assays. In vitro proliferation assays were performed by using non-proliferating PLP_139-151_-specific T cells (see above). Cells were incubated for 16 h with PBS or two different concentrations of PTTH (0.1 mM and 0.5 mM). After treatment, cells were washed and restimulated in 96-well plates in the presence of irradiated splenocytes as APCs (ratio 1:8) and increasing concentrations of PLP_139-151_ peptide (0, 10, 20, or 30 μg/ml). After 48 h, cells were pulsed with 1 μCi/well of ^3^H-thymidine (dT) and were left in culture for further 18 h.
2. Ex vivo proliferation assay. Draining LNs cells were collected from vehicle- or PTTH-treated SJL EAE mice at disease peak. 0.4 × 10^6^ total LN cells were re-stimulated in a 96-well plate with PLP_139-151_ peptide 30 μg/ml. After 48 h, cultures were pulsed for 18 h with 1 μCi/well of ^3^H-dT.

For both 1. and 2., samples were harvested after 18 h and supplemented with 3 ml of scintillation fluid (Ultima Gold from Perkin-Elmer). ^3^H-dT incorporation by proliferating cells was measured with a β-counter (Perkin-Elmer) and quantified as counts per minute (CPM). For each experiment, a 96-well plate was seeded for ^3^H-dT incorporation assay as explained before, and another identical one, but without ^3^H-dT, for supernatant collection for cytokine quantification.

### Bio-Plex assay for cytokines detection

Supernatants collected from in vitro and  ex-vivo proliferation assays were used for Milliplex cytokine assays (Merck Millipore), following manufacturer’s instructions. Briefly, anti-cytokine conjugated beads were plated in 96-well microtiter plates and then removed by vacuum filtration. Samples were then added, and the plate was incubated for 30 min by mixing at 300 rpm. Bio-Plex cytokine assays were sequentially incubated with the detection antibody and streptavidin-PE; samples were then analyzed immediately using the Bio-Plex array-system. Cytokine concentrations were automatically calculated by Bio-Plex software using a standard curve derived from recombinant cytokine standards.

### In vitro adhesion assays on purified integrin ligands

12-well glass slides were coated for 18 h at 4 °C with purified mouse intercellular cell adhesion molecule (ICAM)-1 or vascular cell adhesion molecule (VCAM)-1 (R&D Systems), 1 μg/ml in PBS. Slides were then blocked with FBS for 10 min at 37 °C. Activated PLP_139-151_-specific T cells were pre-treated for 6 h with PTTH 0.5 mM or 1.0 mM. Cells were then re-suspended at 5 × 10^6^/ml in standard adhesion buffer. 20 μl of cell suspension were added to each well, and cells were left spontaneously adhere on VCAM-1 or ICAM-1 for 20 min at 37 °C. After washing, adherent cells were fixed in glutaraldehyde 1.5% in PBS and counted by computer-assisted enumeration [9].

### Intravital microscopy in brain pial venules

Intravital microscopy experiments were performed in inflamed brain microcirculation as previously described [39, 46]. Wild-type C57Bl/6 mice were injected intraperitoneally with 12 μg lipopolysaccharide (LPS) (Sigma-Aldrich) 5–6 h before starting the experiment. Animals were anesthetized and a heparinized PE-10 catheter was inserted into the right common carotid artery toward the brain. Blood vessels were visualized through the bone using fluorescent dextran as described [39, 46]. 2–3 × 10^6^ activated PLP_139-151_-specific T cells were treated with vehicle or PTTH 1.0 mM for 6 h, labeled with CMFDA (5-chloromethylfluorescein diacetate) or CMTMR (5-(and-6)-(((chloromethyl)benzoyl)amino)tetramethylrhodamine) (Thermo Fisher Scientific), and injected into the carotid artery by a digital pump. Hemodynamic parameters were determined as described [39, 46]. Images were visualized with a silicon-intensified target video camera (VE-1000 SIT; Dage-MTI) and a Sony SSM-125CE monitor. Digitalized video images were analyzed frame by frame using the ImageJ software. Lymphocytes that remained stationary on the venular wall for ≥ 30 s were considered firmly adherent. Rolling and firm arrest fractions were determined as the percentage of cells that rolled or firmly arrested within a given venule on the total number of cells entering the venule.

### Differentiation and analysis of murine Th0, Th1, Th17 and Th2 cells

Resting CD4^+^CD62L^+^ T cells were isolated in a two-step separation from the spleens of 6–8 weeks old C57Bl/6 J mice, using the CD4 + T Cell Isolation Kit and the CD62L (L-selectin) MicroBeads (both from Miltenyi Biotech), following manufacturer’s instructions. T cells were incubated for 8 h in complete medium with vehicle (PBS) or PTTH 0.5 mM. Cells were then washed and seeded on plate-bound anti-CD3/anti-CD28 antibodies (1 µg/ml and 2 µg/ml, respectively—BioLegend) in complete medium. The following cytokines and blocking antibodies were also added to induce Th0, Th1, Th2 or Th17 cells, respectively: no cytokines for Th0 cells; interleukin-12 (IL-12) 20 ng/ml + anti-IL-4 antibody 5 µg/ml (BD Pharmingen) for Th1 cells; IL-4 40 ng/ml + anti-interferon gamma (IFN-γ) antibody 5 µg/ml (BD Pharmingen) for Th2 cells; IL-1β 10 ng/ml + IL-6 50 ng/ml + IL-23 10 ng/ml + transforming growth factor-beta (TGF-β) 0.5 ng/ml + anti-IL-4 and anti-IFN-γ antibodies 5 µg/ml for Th17 cells. Cytokines were purchased from ImmunoTools (IL-1β, IL-6 and TGF-β), Miltenyi Biotech (IL-12 and IL-23) or BioLegend (IL-4). For cell proliferation analysis, cells were stained with the fluorescent dye CellTraceViolet (CTV, Thermo Fisher Scientific) before activation, following manufacturer’s instructions. After 3 days, CTV-stained cells were collected, washed, and acquired. To analyze cytokine production, cells were re-stimulated for 5 h with brefeldin A (BFA) 10 µg/ml, ionomycin 1 µg/ml and phorbol-12-myristate-13-acetate (PMA) 50 ng/ml. Cells were then fixed and permeabilized with fixation buffer and intracellular staining permeabilization wash buffer (BioLegend) and stained with the following antibodies: APC anti-mouse IFN-γ, APC/Cy7 anti-mouse tumor necrosis factor alpha (TNF-α) and PE/Dazzle 594 anti-mouse granulocyte–macrophage colony-stimulating factor (GM-CSF) antibodies (Th0 cells); APC anti-mouse IFN-γ antibody (Th1 cells); APC anti-mouse IL-13 antibody (Th2 cells); PE anti-mouse IL-17A and Alexa647 anti-mouse IL-17F antibodies (Th17 cells) (all antibodies from BioLegend). All samples were acquired on a BD LSRFortessa and analyzed using FlowJo (BD Biosciences).

### EAE induction and in vivo treatment with PTTH

For relapsing–remitting (RR)-EAE, SJL female mice were immunized s.c. with 300 μg of PLP_139-151_ peptide in 200 μl emulsion as described above. For chronic EAE, C57Bl/6 female mice were immunized s.c. with 300 μg myelin oligodendrocyte glycoprotein (MOG)_35–55_ peptide in 200 μl emulsion. In both models, 20 ng of pertussis toxin were injected intraperitoneally (i.p.) at the day of immunization and after 48 h. To evaluate the clinical benefit of PTTH, mice were treated i.p. with 30 mg/mouse/day of PTTH or saline (0.9% NaCl; control animals). In the pre-clinical setting, mice received a daily injection of PTTH or corresponding volume of saline starting 5 days post-immunization, before disease onset. In the therapeutic protocol, PTTH or saline were administered i.p. after the initial peak of the disease (day + 18 post-immunization) in the RR-EAE model, whereas treatment started at the beginning of the chronic phase (day + 23 post-immunization) in the chronic EAE model. In both pre-clinical and therapeutic settings, mice were treated for 20 consecutive days. Clinical score was recorded daily as previously described [2].

### Neuropathology

SJL mice from pre-clinical treatment experiments were sacrificed at disease peak and perfused with PBS and 4% paraformaldehyde (PFA, Vetrotecnica). Lumbar spinal cords (SCs) were collected, put overnight in PFA 4%, and for a further overnight in a 30% saccharose water solution. SCs were finally embedded in tissue freezing medium (Leica Biosystems), frozen in dry ice, and stored at − 80 °C. 10 µm sections were histologically processed using standard hematoxylin/eosin (H/E) staining for detection of inflammatory infiltrates. Images were acquired using the Axio Imager Z2 (Zeiss, Germany) and quantification of infiltrated areas was performed on every fifth section of the SCs using the ImageJ software.

### Differentiation and analysis of human Th1, Th17 and induced regulatory T (iTregs) cells

Peripheral blood mononuclear cells (PBMCs) were isolated by density gradient centrifugation with Lymphoprep (Proteogenix), from buffy coats obtained from the Transfusion Medicine Department of the Medical University of Graz. Naive CD4^+^ T cells were purified from PBMCs using the MagniSort human CD4 naive enrichment kit (Thermo Fisher Scientific), following manufacturer’s instructions. T cells were activated in vitro for 5 days in X-VIVO 15 medium (Lonza) with plate-bound anti-CD3/anti-CD28 antibodies (1 µg/ml and 2 µg/ml, respectively—BioLegend), in the presence of vehicle (PBS) or PTTH 1.0 mM. The following cytokines were also added to induce Th1, Th17, or iTreg cells, respectively: IL-12 10 ng/ml for Th1 cells; IL-1β 10 ng/ml + IL-6 20 ng/ml + IL-23 100 ng/ml + TGF-β 1 ng/ml for Th17 cells; IL-2 10 ng/ml + TGF-β 5 ng/ml for iTregs cells. Cytokines were purchased from ImmunoTools (IL-6, IL-23, TGF-β and IL-2), Peprotech (IL-1β) or R&D Systems (IL-12). For cell proliferation analysis, cells were stained with CTV, collected after 5 days of culture, washed, and acquired. To analyze cytokine production, cells were re-stimulated with PMA/BFA/ionomycin, fixed and permeabilized as described above for mouse T cells. Cells were then stained with the following antibodies: Alexa488 anti-human IFN-γ and PE/Dazzle 594 anti-human GM-CSF antibodies (Th1 cells); APC/Cy7 anti-human IL-17A and PE/Dazzle 594 anti-human GM-CSF antibodies (Th17 cells) (all antibodies from BioLegend). To analyze Treg induction, cells were collected after 5 days of culture and stained with the following antibodies: PE anti-human CD25 and Alexa647 anti-human-CD127 (both antibodies from BioLegend). Cells were then fixed and permeabilized with the eBioscience™ Foxp3/Transcription Factor Staining Buffer Set (Thermo Fisher Scientific) and stained with the Alexa488 anti-mouse/rat/human Forkhead box P3 (FOXP3) antibody (BioLegend). All samples were acquired on a BD LSRFortessa and analyzed using FlowJo.

### MS patient recruitment and analysis

All participants included in this study underwent clinical examination at the MS outpatient clinic of the Department of Neurology, Medical University of Graz. Participants (n = 15; Table 1) met the following criteria: (a) age > 18 years at baseline; (b) diagnosed with clinically isolated syndrome (CIS) suggestive of MS or with MS according to diagnostic criteria applicable at the time of presentation [12], (c) did not receive disease-modifying treatment and/or cortisone for at least 6 months prior to blood collection. PBMCs were isolated from 24 ml blood by density centrifugation, washed, counted, and resuspended in RPMI medium + FBS:DMSO (15 ml:3.7 ml) mixture. Cells were then divided in cryogenic vial (5–8 × 10^6^ cells/vial) and gradually frozen at − 80 °C for at least 24 h. Cell vials were finally transferred into a cryo tank (liquid nitrogen vapor phase) and stored until use. At the time of the experiments, PBMCs were thawed, washed, counted, and recovered in X-VIVO medium for 1 h at 37 °C. Cells were resuspended in fresh X-VIVO medium and activated with plate-bound anti-CD3/anti-CD28 antibodies as above, in the presence of PBS or PTTH 1.0 mM. After 5 days, cells were collected, washed, re-stimulated as above with BFA/PMA/ionomycin, fixed, permeabilized, and stained with Alexa488 anti-human IFN-γ + PE/Dazzle 594 anti-human GM-CSF + APC/Cy7 anti-human IL-17A + BV421 anti-human IL-10 (BioLegend) + APC anti-human TNF-α (BioLegend) antibodies. Samples were acquired and analyzed as above.

**Table 1:** Demographic and clinical data of MS patients recruited in the study

Number of pwMS	15
Number (%) females	10 (66.7%)
Age—years, mean (SD)	40.3 (6.4)
Age at disease onset—years, mean (SD)	30.6 (9.0)
Disease course, n (CIS/RRMS/PMS)	6/4/5
Disease duration—years, mean (SD)	9.6 (7.6)
EDSS in remission, years, median (IQR)	1.9 (2.3)

*pwMS* persons with multiple sclerosis, *CIS* clinically isolated syndrome, *RRMS* relapsing remitting multiple sclerosis, *PMS* progressive multiple sclerosis, *EDSS* expanded disability status scale, *SD* standard deviation, *IQR* inter quartile range

### Statistics

Statistical analyses for metabolomics and phosphoproteomics studies were performed as described above. For in vitro, ex-vivo, and in vivo experiments, statistical analyses were performed using GraphPad Prism 10 (GraphPad Software Inc.). Data are presented as mean values ± standard deviation (SD) or standard error of the mean (SEM). The statistical tests performed for each experiment are reported in the figure legend, and a *p*-value < 0.05 was considered significant.

## Results

### Alteration of CoA metabolism in PLP_139-151_-specific T cells

To investigate the metabolic alterations associated with the neuroinflammatory potential of autoreactive myelin-specific T cells, we analyzed the metabolome of actively-proliferating murine PLP_139-151_-specific encephalitogenic T cell lines and compared it with the one of resting CD4^+^ T cells freshly-isolated from LNs of naïve SJL mice. PLP_139-151_-specific T cell lines can induce autoimmune neuroinflammation once injected in recipient animals, representing an ideal model of autoreactive T cells [25].

**Fig. 1:**
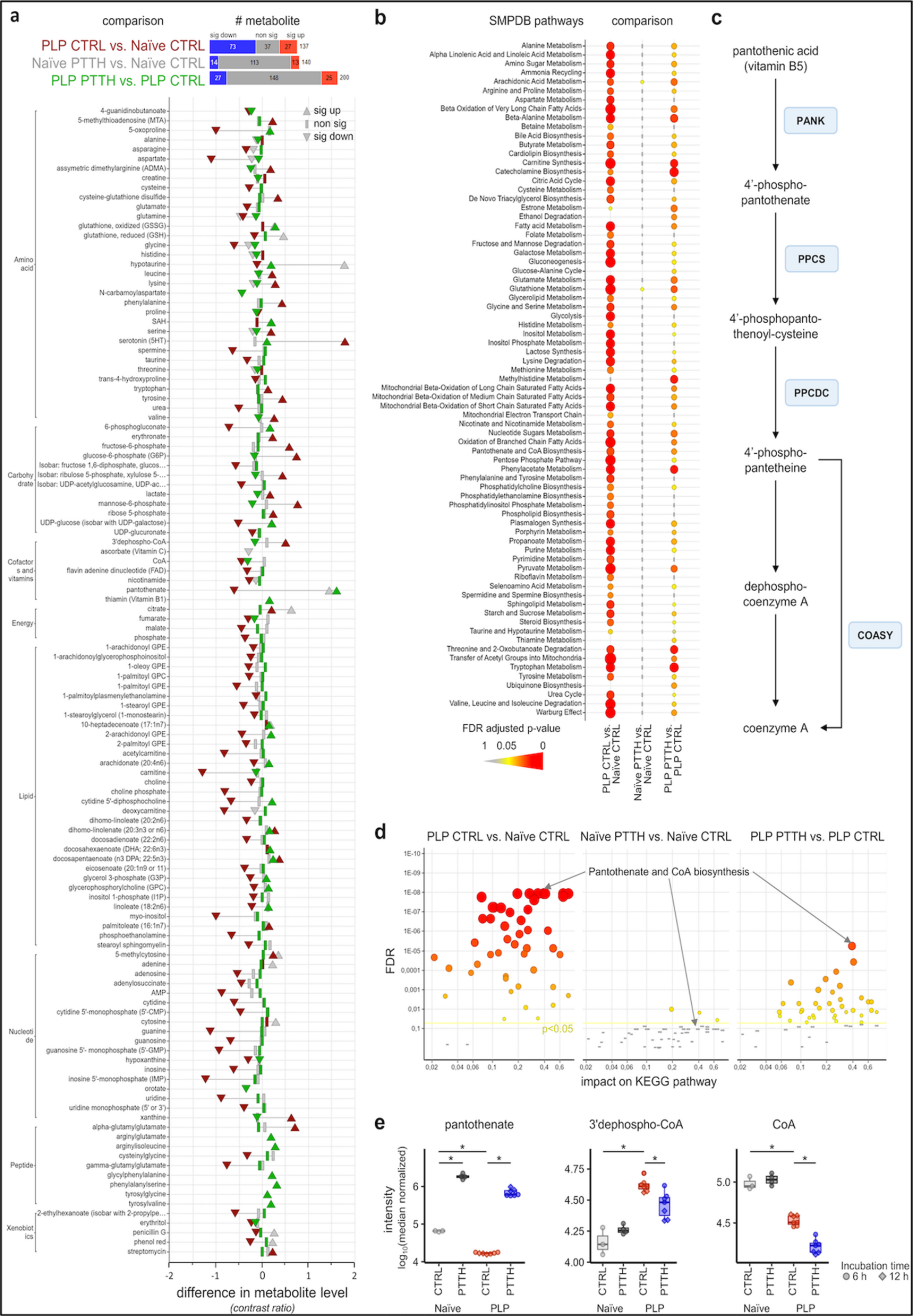
Altered CoA synthesis in actively-proliferating PLP_139-151_ specific encephalitogenic T cells is restored by PTTH treatment. Actively-proliferating encephalitogenic T cells (PLP) were treated with either vehicle (CTRL) or PTTH 1.0 mM (PTTH) for 6 or 12 h prior to metabolomics analysis, and were compared to resting T cells (Naïve) with/without PTTH. **a** Dumbbell plot of metabolites with any significant change in one of the three comparisons of interest. The plot shows the strength of metabolic changes along the x-axis and the metabolite name and class on the y-axis with thin lines connecting the same metabolite. The FDR adjusted significance (*p* < 0.05) is encoded in shapes indicating also direction (up- or down-regulated). The bar chart beside the comparison (top) counts the number of significantly changed metabolites. **b** Results from the quantitative pathway enrichment analysis against SMPDB for each of the three comparisons of interest are plotted on the x-axis and the pathway names in alphabetic order on the y-axis. Non-significant pathways are represented as bars (FDR adjusted *p* > 0.05) and significant ones as circles. The FDR adjusted significance is also encoded into gradually increasing dot size and intensifying color with redder/larger for more significantly impacted pathways. **c** The CoA synthesis pathway. PANK: pantothenate kinase. PPCS: phosphopantothenate-cysteine ligase. PPCDC: phosphopantothenoylcysteine decarboxylase. COASY: coenzyme A synthase. **d** Quantitative pathway topological analysis results for each of the three comparisons of interest plotting the pathway impact on the x-axis against the FDR adjusted significance on the y-axis with the same shape, size and color settings as in **b**. **e** Box plots of different metabolites with middle lines marking the 50th percentile, box edges marking the 25th and 75th percentile and whiskers marking the 1.5 interquartile range. *p < 0.05 by linear mixed models (see ‘Methods‘ section for details). Data in **a–d** are from N = 3 (Naïve CTRL), N = 4 (Naïve PTTH), and N = 7 (PLP CTRL and PTTH) independent samples

By performing broad, unbiased metabolomics, we found the levels of most metabolites to be significantly altered in encephalitogenic T cells (Fig. 1a, Fig. S1c, Supplementary data 1). This drastic metabolic derailment was also obvious in unsupervised multivariate PCA analysis of global metabolic changes (Fig. S1a) and was confirmed to be significant in a supervised PLS-DA analysis (Fig. S1b). A quantitative pathway enrichment analysis showed that encephalitogenic T cells displayed altered engagement of energy-provision pathways such as the pentose phosphate pathway (PPP), glycolysis, TCA cycle, mitochondrial oxidation of short, medium and long chain saturated fatty acids, mitochondrial electron transport chain activity, as well as many amino acid pathways (Fig. 1b). In particular, while resting CD4^+^ T cells showed higher levels of TCA cycle intermediates, such as fumarate and malate, activated encephalitogenic T cells displayed increased levels of free glucose, glycolysis intermediates such as glucose-6-phosphate and fructose-6-phosphate, PPP intermediates such as ribulose-5-phosphate, and lactate (Fig. 1a, Supplementary data 1). Together with a generalized altered level of Warburg metabolism highlighted by the pathway enrichment analysis (Fig. 1b), these data show the expected metabolic shift of autoreactive T cells towards aerobic glycolysis during active proliferation [5, 19].

Interestingly, compared to resting T cells, one of the pathways found to be altered in encephalitogenic T cells was the ‘Pantothenate and CoA biosynthesis’ pathway (Fig. 1b–e). Strikingly, this pathway displayed one of the highest significances and pathway impact scores in a pathway topology analysis (Fig. 1d). In actively-proliferating PLP_139-151_-specific T cells, the CoA synthesis pathway was characterized by a decrease in the levels of pantothenate, paired to an accumulation of 3’-dephospho-CoA and lower levels of free CoA (Fig. 1a and e, Supplementary data 1). This suggests a potential block of CoA synthesis at the level of 3’-dephospho-CoA in proliferating encephalitogenic T cells.

### The CoA precursor pantethine (PTTH) restores CoA synthesis and displays immunomodulatory properties in PLP_139-151_-specific T cells

Considering the potential block of the CoA synthesis pathway in actively-proliferating encephalitogenic T cells and its high pathway impact score (Fig. 1d), we decided to evaluate the effect of pantethine (PTTH) on encephalitogenic T cell metabolic profile. The low molecular weight thiol PTTH is the stable homodimeric form of pantetheine (Fig. S2a) and was previously shown to increase CoA levels both in vitro and in vivo [10, 43, 60]. We first confirmed that treatment with PTTH rapidly increased total CoA levels in encephalitogenic T cell lines (Fig. S2b). We then assessed the effect of PTTH on the metabolome of actively-proliferating encephalitogenic T cell after 6 h–12 h treatment, which was highly significant and profound. Importantly, PTTH had in contrast rather weak effects on resting T cells with almost no significant impact on the investigated pathways (Fig. 1a, b, and d, Fig. S1, Supplementary data 1). As expected, in both resting and encephalitogenic T cells treated with PTTH we detected a strong elevation in pantothenate, which is generated from PTTH and is the primary precursor for CoA synthesis. Moreover, in PLP_139-151_-specific T cells, intracellular levels of 3’dephospho-CoA were significantly reduced upon PTTH treatment, suggesting a partial restoration of the CoA synthesis pathway (Fig. 1a and e, Supplementary data 1). Surprisingly, our metabolomics analysis also showed a reduction in free CoA levels in PTTH-treated encephalitogenic T cells (Fig. 1a and e, Supplementary data 1). We hypothesize that this reduction at 6 h and 12 h post-treatment may be caused by the fast consumption of newly-produced CoA in the numerous reactions in which this molecule is involved [27]. Apart from refueling the CoA synthesis pathway, PTTH remodeled the overall metabolic profile of proliferating PLP_139-151_-specific T cells, mainly impacting lipid- and amino acid/peptide levels (Fig. 1a, Supplementary data 1). Notably, the CoA synthesis pathways scored as the most significant one altered by PTTH treatment in encephalitogenic T cells (Fig. 1d).

**Fig. 2:**
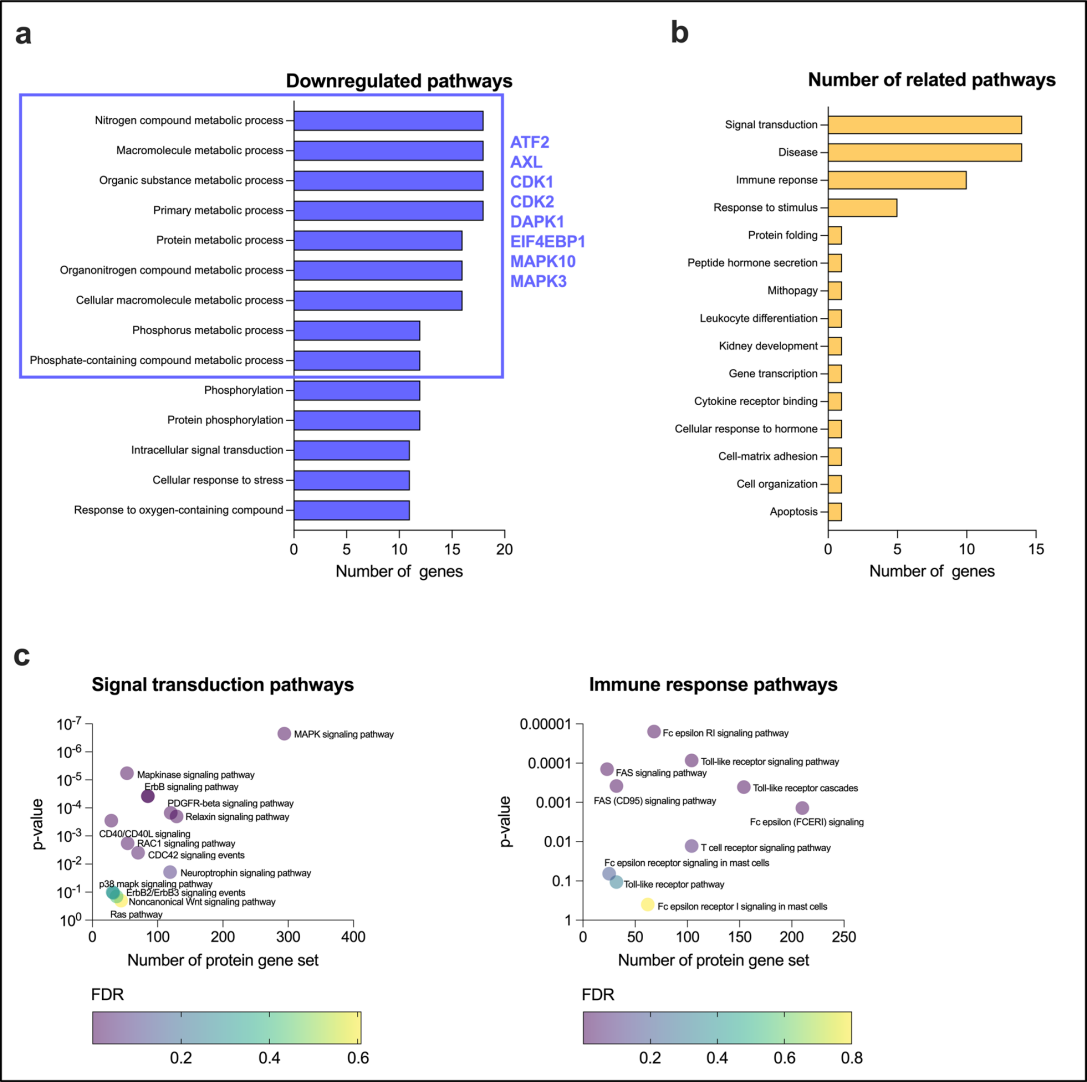
Phosphoproteomics analysis revealed an immunomodulatory effect of PTTH in autoreactive encephalitogenic T cells. Actively-proliferating encephalitogenic T cells were treated with PTTH 1.0 mM for 6 h and analyzed in outsourcing for total protein expression and phosphorylation by Kinexus (see Supplementary data 2 for protein dataset). A bioinformatics analysis was then performed as described in the “Materials and methods” section. **a** Results from Gene GO analysis on down-regulated proteins following PTTH treatment are shown. **b** The bar plot represents the number of related pathways for each group calculated as described in the “Results” section. **c** Pathways included in the “Signal transduction” and “Immune response” group are shown. Data are from one representative encephalitogenic T cell line

Recent reports suggested a potential immunomodulatory activity of PTTH [1, 31]. Yet, no studies previously investigated whether PTTH may affect autoreactive T cell pathogenic potential in autoimmune neuroinflammation. We thus evaluated the overall immunomodulatory impact of PTTH in actively-proliferating PLP_139-151_-specific T cells by performing phosphoproteomics analysis. We found 30 significant proteins with a Z-ratio of ± 1.1 in terms of expression or phosphorylation differences between encephalitogenic T cells treated or not with PTTH (Supplementary data 2—‘Proteins’). GO enrichment analysis was performed on up- or down-regulated proteins separately (Supplementary data 2—‘GO enrichment’). Interestingly, the bioinformatic analysis reported that several metabolic pathways were downregulated following PTTH treatment, supporting the data described above. All these pathways shared 8 proteins (ATF2, AXL, CDK1, CDK2, DAPK1, EIF4EBP1, MAPK10, and MAPK3), which were selected as a “bioinformatic probe” for the network analysis (Fig. 2a). Next, topological network analysis first led to the identification of a restricted signal transduction sub-network, including several proteins involved in T-cell signaling mechanisms. Further network modular decomposition led to the extraction of a highly interconnected signaling module including six MAPKs (Fig. S3). Reactome enrichment analysis detected 55 enriched pathways, which were manually classified into groups based on their pathway hierarchy (Supplementary data 2—‘Merged network’). Calculating the number of related pathways for each group, we revealed that, despite the broad cellular effects of PTTH, the majority of enriched pathways following the PTTH treatment were involved in signal transduction (Fig. 2b), with the best-enriched pathway represented by MAPK signaling pathway involved in T cell proliferation and survival (Fig. 2c) [17]. Among the signal transduction pathways modified by PTTH treatment, we also identified several pathways involved in leukocyte integrin-dependent adhesion and motility, including the “RAC1 signaling pathway”, “RAS pathway”, and “CDC42 signaling events” (Fig. 2c). In addition, we found that immune response-related pathways involved in T cell activation, such as Toll-like receptor, T cell receptor and FAS signaling, were also altered by the PTTH treatment (Fig. 2c). Collectively, these data suggest that CoA fueling with PTTH have a strong immunomodulatory effect on PLP_139-151_-specific encephalitogenic T cells, significantly affecting their activation, proliferation and migration.

### PTTH inhibits the pathogenic potential of PLP_139-151_-specific T cells

**Fig. 3:**
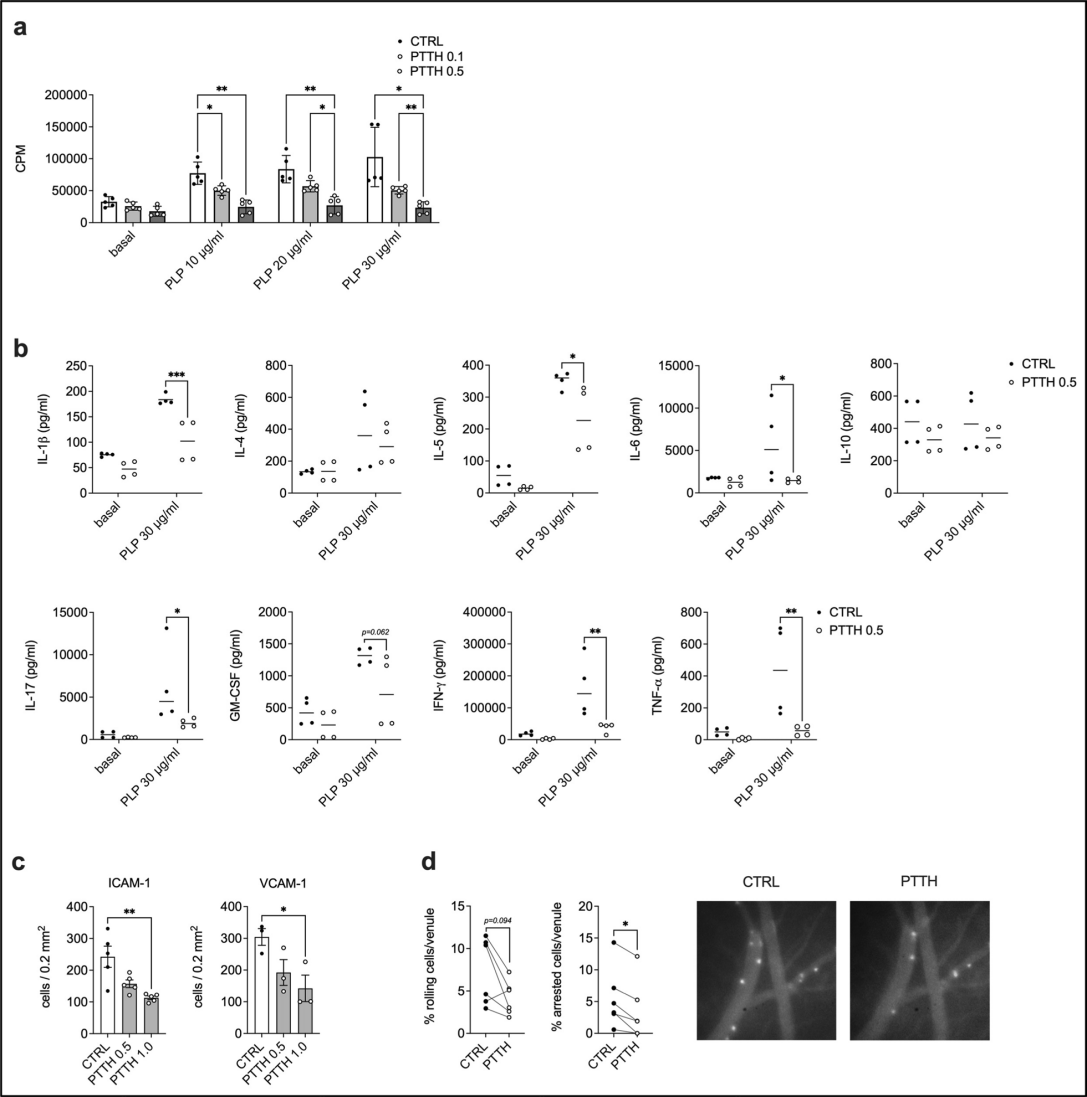
Pantethine inhibits key pathogenic features of encephalitogenic T cells in vitro. **a** Non-proliferating encephalitogenic T cells were treated with vehicle (CTRL) or increasing mM concentrations of pantethine (PTTH) for 16 h, washed, and re-stimulated for 3 days with APCs and PLP_139–151_. Data are the mean ± SD of N = 5 independent cell lines. *p < 0.05 and **p < 0.01 by 2-way Anova with Tukey’s test for multiple comparisons. **b** Cytokine quantification in supernatants collected from the experiments performed in **a**. N = 4 independent cell lines. *p < 0.05, **p < 0.01, and ***p < 0.001 by 2-way Anova with Šídák’s test for multiple comparisons. For better clarity, only comparisons between CTRL and the corresponding PTTH conditions are show. **c** Activated PLP_139-151_-specific T cells were treated with vehicle (CTRL) or increasing mM concentrations of PTTH for 6 h. Cells were then left to spontaneously adhere on glass slides pre-coated with ICAM-1 of VCAM-1. Data are the mean ± SEM of 5 (ICAM-1) or 3 (VCAM-1) independent experiments. *p < 0.05 by Friedman test with Dunn’s test for multiple comparisons. **d** Left—Activated encephalitogenic T cells were treated with vehicle (CTRL) or PTTH 1.0 mM and injected in LPS-treated mice to analyze their adhesive potential in vivo. *p < 0.05 by Wilcoxon test. Data obtained analyzing 6 venules from 3 independent experiments are shown. Right—representative images of brain pial venules upon injection with CTRL or PTTH-treated cells. Cells are the white dots in the blood vessels

To confirm the phosphoproteomics observations in functional assays, we first analyzed the effect of PTTH on the antigen-induced proliferation of PLP_139-151_-specific encephalitogenic T cells. To more closely mirror an in vivo setting, where resting antigen-specific T cells are (re-)activated by APCs in secondary lymphoid organs or peripheral tissues, we evaluated the impact of PTTH on the re-activation capacity of non-proliferating, resting encephalitogenic T cell lines. In this setting, we found that pre-treatment with PTTH strongly inhibited the antigen-specific proliferation of PLP_139-151_-specifc T cell lines upon restimulation in the presence of PLP_139-151_ and APCs (Fig. 3a), confirming that PTTH blocks APC-induced antigen-specific T cell re-activation in vitro. We also observed that treatment with PTTH drastically reduced the secretion of several pro-inflammatory cytokines by proliferating encephalitogenic T cell lines, including IFN-γ, IL-17, IL-6, TNF-α and GM-CSF (Fig. 3b). As these pro-inflammatory cytokines play a key role in the development of neuroinflammation in both EAE and MS [4], our results confirm that PTTH may inhibit encephalitogenic T cell pathogenicity. Interestingly, PTTH also inhibited the production of IL-5 and to a lesser extent IL-10, indicating a more general immunomodulatory effect on the PLP_139-151_- specific T cell lines (Fig. 3b). Strikingly, when evaluating the effect of PTTH on pan-T cell receptor (TCR)-activated T cells, we found no significant impact of PTTH treatment on the proliferation and cytokine production of several murine T cells subsets (Th0, Th1, Th2 and Th17 cells) (Fig. S4). These results were unexpected, and may suggest a preferential effect of PTTH on antigen-specific T cell activation.

The ability of encephalitogenic T cells to adhere to inflamed CNS pial venules and to migrate in the surrounding CNS parenchyma is a crucial event in the induction of inflammatory responses against the neuronal myelin sheath. In particular, integrin-mediated T cell adhesion is an essential step in this process [47]. It was previously shown that PTTH treatment down-regulates platelet hyper-adhesion in an animal model of cerebral malaria, suggesting that it may impact cell adhesion to the activated endothelium [38]. Moreover, our phosphoproteomics data indicate that PTTH may affect T cell adhesiveness, migration, and integrin signaling. We thus investigated the effect of PTTH treatment on the integrin-dependent adhesion of encephalitogenic T cells on purified ICAM-1 and VCAM-1 in vitro. We found that pre-treatment with PTTH significantly reduced the spontaneous adhesion of activated encephalitogenic T cells on both integrin ligands in a dose-dependent manner, compared to untreated cells (Fig. 3c). As ICAM-1 and VCAM-1 are two major integrin ligands expressed by the inflamed CNS endothelium in EAE and MS [47, 48], these data further support the concept that PTTH may inhibit encephalitogenic T cell pathogenicity.

We next sought to evaluate whether PTTH could also block the adhesion of encephalitogenic T cells in vivo in inflamed CNS pial venules. Pial venules represent a preferential entry point for pathogenic T cells in the CNS during the development of EAE, and we and others have previously demonstrated that encephalitogenic T cell adhesion in CNS pial venules is partially mediated by integrins and their endothelial counter-ligands [23, 24, 47, 49]. We thus assessed encephalitogenic T cell adhesiveness in brain pial venules of LPS-treated mice, an experimental model that mimics the early inflammatory condition during the pre-clinical phase of EAE, when integrins are essential for T cell adhesion to the CNS vasculature [39]. We observed that PTTH-pre-treated encephalitogenic T cells displayed a significant reduction of their rolling and arrest on inflamed brain vessels, compared to control cells (Fig. 3d), clearly demonstrating an inhibitory effect of CoA fueling with PTTH on integrin-mediated adhesion also in vivo.

### PTTH inhibits EAE development and ameliorates disease severity in established EAE

**Fig. 4:**
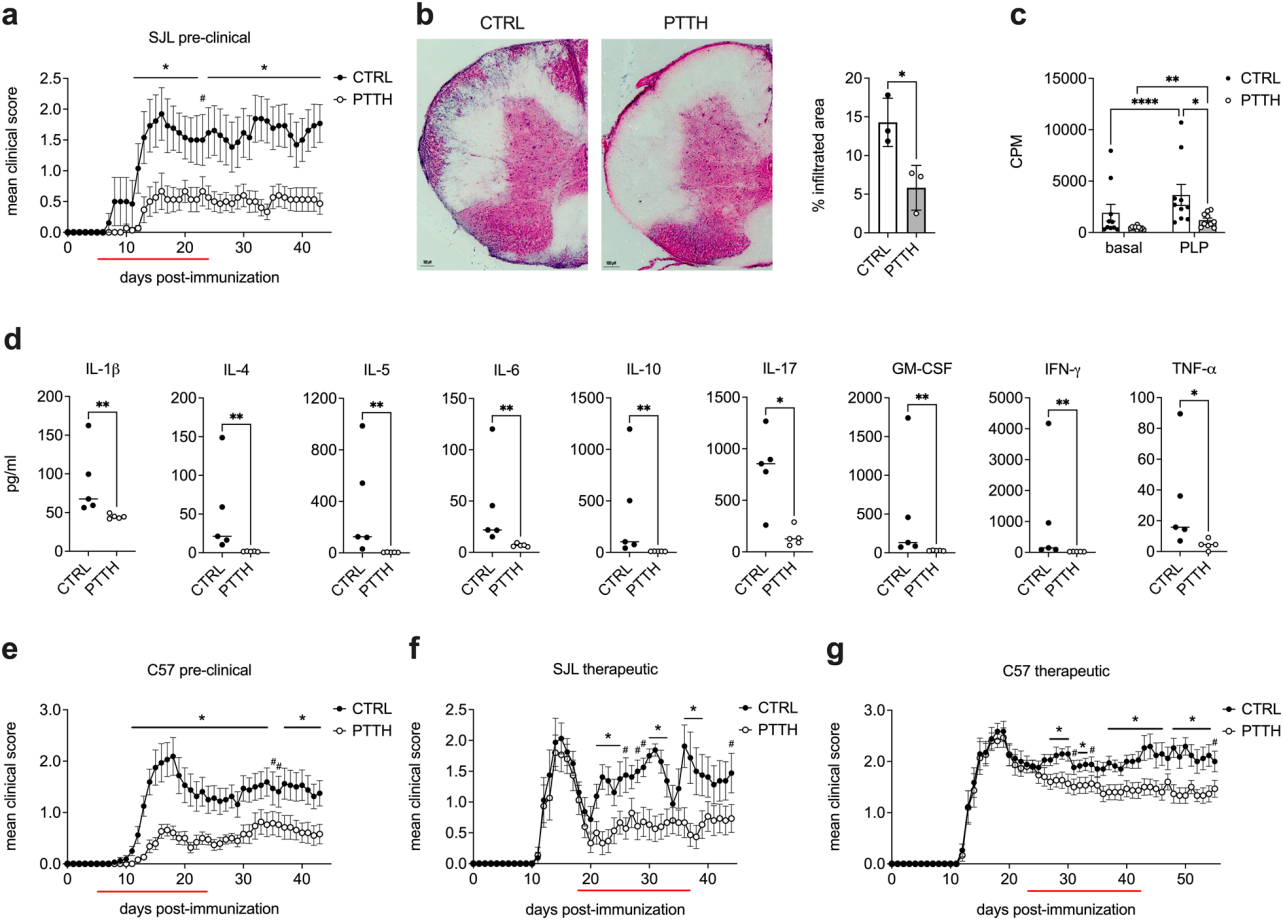
Pantethine affects RR-EAE and chronic EAE development and has immunomodulatory properties in vivo. **a** SJL EAE-immunized mice were treated i.p. with saline (CTRL) or 30 mg/day of pantethine (PTTH) from day + 5 post-immunization for 20 consecutive days (red line). *p < 0.05 and ^#^*p* < 0.1 by two-tailed Student’s t-test. Data are the mean ± SEM of two independent experiments. See Table 2 for quantification of the results. **b** Left: representative image of H/E staining of SC sections from vehicle- (CTRL) or PTTH-treated mice collected at disease peak from SJL mice undergoing a pre-clinical treatment regime. Right: infiltrates were identified in 3 CTRL and 3 PTTH-treated mice. The % of area showing immune cell infiltration on the total SC area is shown. *p < 0.05 by unpaired t-test. **c** Total LN cells were collected at disease peak from CTRL and PTTH-treated mice, and re-stimulated *ex-vivo* with PLP_139-151_ peptide. Data are the mean ± SD of 10 mice/condition from two independent experiments. *p < 0.05 and **p < 0.01 and ****p < 0.0001 by 2-way Anova with Šídák’s test for multiple comparisons. (**d** The amount of the displayed cytokines was quantified in the supernatants of proliferation experiments in **c**, analyzing their levels in PLP_139-155_-restimulated samples. Data represent N = 5 mice/condition from one representative experiment. *p < 0.05 and **p < 0.01 by Mann–Whitney test. **e** C57Bl EAE-immunized mice were treated i.p. with saline (CTRL mice) or 30 mg/day of PTTH from day + 5 post-immunization for 20 consecutive days (red line). *p < 0.05 and ^#^*p* < 0.1 by two-tailed Student’s t-test. Data are the mean ± SEM of two independent experiments. See Table 2 for quantification of the results. **f, g** SJL (**f**) or C57Bl (**g**) EAE-immunized mice were treated i.p. with saline (CTRL) or 30 mg/day of PTTH from day + 18 post-immunization (SJL mice) or from day + 23 post-immunization (C57Bl/6 mice) for 20 consecutive days (red line). *p < 0.05 and ^#^*p* < 0.1 by two-tailed Student’s t-test. Data are the mean ± SEM of two independent experiments. See Table 3 for quantification of the results

In light of the metabolic and immunomodulatory effect of CoA fueling on PLP_139-151_-specific encephalitogenic T cells in vitro, we sought to test the clinical potential of metabolic intervention with PTTH in EAE, an autoimmune inflammatory disease of the CNS that represents the animal model of human MS. We first found that pre-clinical treatment with PTTH reduced the incidence and the severity of RR-EAE in SJL mice, with a drastic reduction of maximal clinical score as well as cumulative score compared to control animals (Fig. 4a, Table 2). Importantly, the protective effect of PTTH was maintained even after the suspension of the treatment (Fig. 4a). H/E staining of lumbar SC sections also confirmed fewer inflammatory infiltrates in the SC of PTTH-treated mice (Fig. 4b).

**Table 2:** Clinical features of EAE mice treated with PTTH (pre-clinical protocol)

	Incidence	Disease onset (dpi)	Mean maximum score	Mean cumulative score
SJL mice (RR-EAE)				
CTRL (N = 13)	100%	12.5 ± 3.1	2.7 ± 1.2	53.9 ± 44.4
PTTH (N = 15)	66.7% (10/15)	14.7 ± 3.8	1.3 ± 1.2 (**)	16.5 ± 19.2 (**)
C57Bl mice (chronic EAE)				
CTRL (N = 16)	100%	11.9 ± 1.6	2.6 ± 1.3	47.2 ± 31.9
PTTH (N = 19)	100%	16.9 ± 6.3 (****)	1.7 ± 0.7 (*)	17.2 ± 14.5 (***)

Data are the mean ± SD (N = total number of mice from two independent experiments). *p < 0.05, **p < 0.01, ***p < 0.001, and ****p < 0.0001 by Mann–Whitney test, compared to the corresponding CTRL condition. Dpi: days post-immunization

We next investigated the effect of PTTH treatment on the activation and pathogenicity of T lymphocytes in vivo. To this purpose, we isolated at disease peak total LN cells from SJL EAE mice treated in a pre-clinical regime with vehicle or PTTH, analyzing their proliferative capacity and cytokine production ex-vivo. As expected, we found that LN cells from PTTH-treated mice displayed a strongly reduced proliferative capacity after ex-vivo re-stimulation with PLP_139-151_, when compared to cells isolated from control animals (Fig. 4c). Furthermore, as observed in vitro for PLP_139-151_-specific encephalitogenic T cell lines, cells isolated from PTTH-treated EAE mice produce significantly lower amounts of pro-inflammatory cytokines crucial for EAE and MS development, such as IL-6, IL-17, IFN-γ and GM-CSF [4], upon ex-vivo re-stimulation, compared to LN cells from control mice (Fig. 4d). However, PTTH had a more generalized immunomodulatory effect in vivo, also reducing the production of several anti-inflammatory cytokines such as IL-10, IL-5, and IL-4 (Fig. 4d). These results clearly indicate that PTTH treatment suppressed T cell-mediated inflammation in EAE mice, and confirm the immunomodulatory effect of PTTH in vivo.

We then tested PTTH in a model of chronic EAE in C57Bl/6 mice, where PTTH significantly delayed disease onset and reduced disease severity. As in SJL mice, the protective effect was maintained after the suspension of the treatment (Fig. 4e; Table 2). Finally, considering a potential translational impact of our results in humans, we tested PTTH in a therapeutic setting, by treating mice after the initial peak of disease in RR-EAE (SJL mice) or at the beginning of the chronic phase in chronic EAE (C57Bl/6 mice). Notably, we found that therapeutic PTTH administration significantly ameliorated disease severity in RR-EAE, with reduced relapses and significantly lower cumulative score and mean maximum score after the beginning of the treatment (Fig. 4f; Table 3). Similarly, in chronic EAE, the cumulative score was reduced after treatment with PTTH, compared to untreated mice (Fig. 4g; Table 3). These results demonstrate that PTTH treatment not only has a protective effect on autoimmune disease development, but can also interfere with established disease mechanisms and ameliorate autoimmune disease progression.

**Table 3:** Clinical features of EAE mice treated with PTTH (therapeutic protocol)

	Disease onset (dpi)	Mean maximum score pre-treatment	Mean cumulative score pre-treatment	Mean maximum score post-treatment	Mean cumulative score post-treatment
SJL mice (RR-EAE)					
CTRL (N = 16)	13.2 ± 1.4	2.6 ± 1.1	12.0 ± 7.1	2.9 ± 1.2	35.2 ± 16.2
PTTH (N = 15)	13.3 ± 1.4	2.4 ± 0.9	10.5 ± 6.2	1.3 ± 1.2 (***)	15.9 ± 18.4 (**)
C57Bl mice (chronic EAE)					
CTRL (N = 17)	13.8 ± 1.7	3.2 ± 0.9	23.0 ± 4.6	2.6 ± 0.8	65.2 ± 16.7
PTTH (N = 15)	13.9 ± 1.5	3.0 ± 0.8	22.0 ± 5.5	2.2 ± 0.5	47.8 ± 15.1 (**)

Data are the mean ± SD (N = total number of mice from two independent experiments). **p < 0.01 and ***p < 0.001 by Mann–Whitney test, compared to corresponding CTRL condition. Dpi: days post-immunization

### PTTH limits the pro-inflammatory potential of MS T cells

**Fig. 5:**
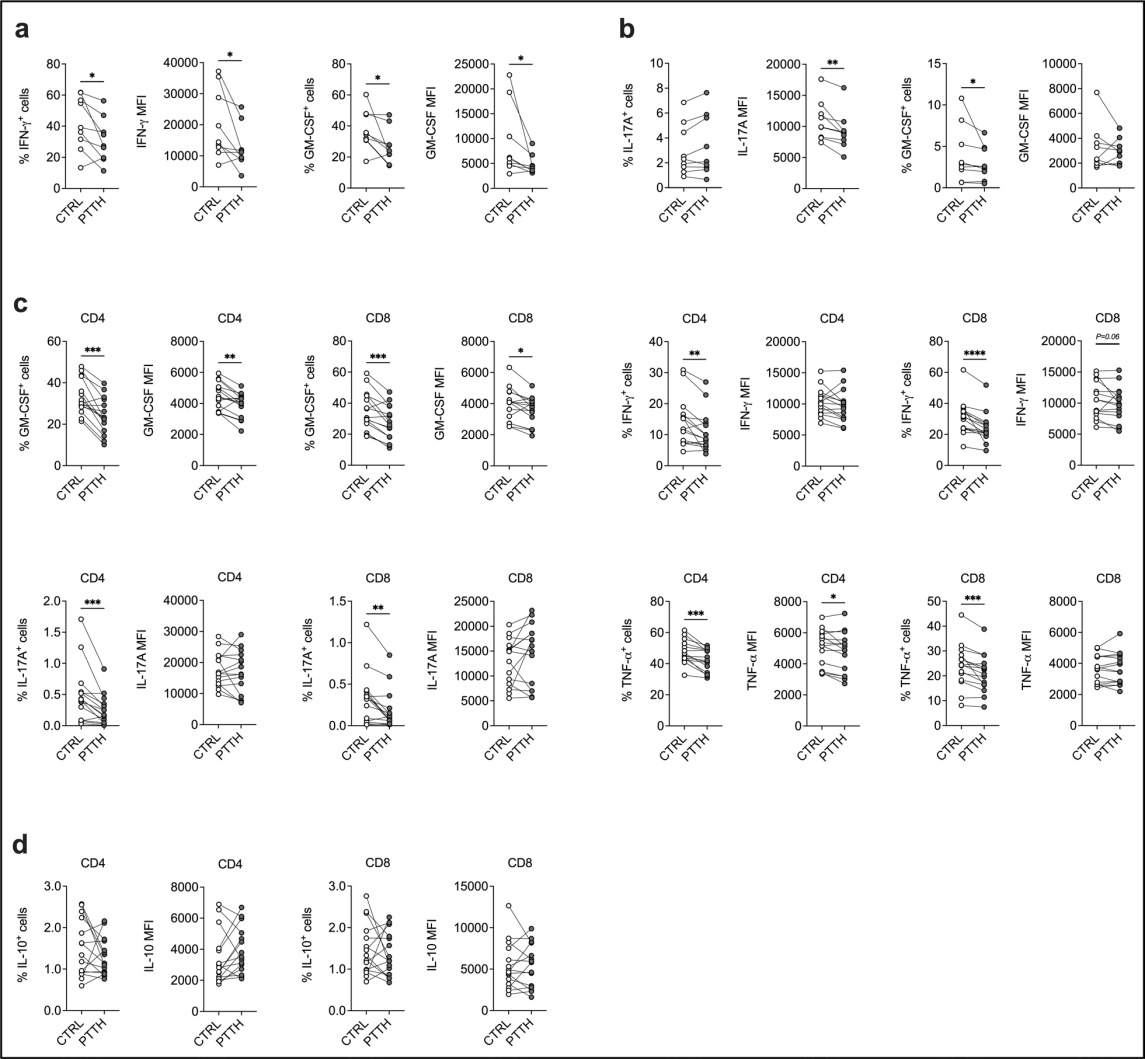
PTTH limits the pro-inflammatory potential of human Th1 and Th17 cells and of T cells from MS patients. **a, b** Naïve CD4^+^ T cells were activated in vitro under Th1- (**a**) or Th17-polarizing conditions (**b**) in the presence of vehicle (CTRL) or PTTH 1.0 mM. Cytokine production was evaluated by intracellular cytokine staining at the end of the culture (day + 5). *p < 0.05 and **p < 0.01 by Wilcoxon test. N = 9 independent donors. (**c, d**) PBMCs from MS patients were activated in vitro with CD3/CD28 antibodies. Cytokine production was evaluated by intracellular cytokine staining at the end of the culture (day + 5). *p < 0.05, **p < 0.01, ***p < 0.001, and ****p < 0.0001 by Wilcoxon test. N = 15 independent patients

To further support the translational potential of PTTH, we analyzed its effect on human Th1 and Th17 cell polarized from naïve CD4^+^ T cell in vitro during pan-TCR activation. First, as for CD3/CD28-activated mouse T cells, we observed that PTTH did not impact neither Th1 nor Th17 cell proliferation in vitro (Fig. S4a). However, in contrast with the results in murine CD4^+^ T cells, PTTH significantly reduced IFN-γ and GM-CSF production by Th1 cells, in terms of percentage of cytokine-producing cells and mean fluorescence intensity (MFI) of the cytokine staining (Fig. 5a). PTTH also reduced IL-17A MFI in Th17 cells, and the percentage of GM-CSF-producing Th17 cells (Fig. 5b), confirming that it may also limit human T cell pro-inflammatory potential. However, incubation with PTTH did not skew Th1 and Th17 towards Tregs cells in vitro, and PTTH did not boost the polarization of Treg cells induced by TGF-β (Fig. S4b).

We then evaluated the impact of PTTH on the production of several cytokines by T cells from 15 MS patients (Table 1). PBMCs from these patients were re-stimulated in vitro with CD3/CD28 antibodies in the presence of PTTH, and cytokine production was analyzed by flow cytometry. Our data show that PTTH reduced the percentage of both CD4^+^ and CD8^+^ T cells producing GM-CSF, IFN-γ, IL-17A, and TNF-α (Fig. 5c). Importantly, production of the anti-inflammatory cytokine IL-10 was not affected (Fig. 5d). These results confirm that CoA fueling with PTTH may also limit T cell pathogenicity in human neuroinflammation.

## Discussion

The activation state and the effector functions of T cells are controlled by the preferential engagement of distinct metabolic pathways [5, 19]. In this study, by using metabolomics, phosphoproteomics and functional approaches, we reported that the CoA synthesis pathway may have a crucial role in the pathogenic potential of myelin-specific autoreactive encephalitogenic T cells, which represent major players in the pathogenesis of EAE and MS. To our knowledge, this is the first report suggesting alteration of such a metabolic pathway in autoreactive T cells. Our metabolomics analysis demonstrated that, as expected, encephalitogenic T cell lines shift their metabolism to aerobic glycolysis and anabolic metabolism when activated by antigen presentation. Strikingly, we also observed a potential brake in the CoA synthesis pathways, with accumulation of 3’-dephospho-CoA and reduced levels of free CoA in the cells. The reason for this alteration is unclear, and a potential explanation may be a reduced uptake or production of pantothenic acid by activated T cells. However, considering the accumulation of 3’-dephospho-CoA, this scenario is unlikely, and a more probable explanation is a reduced expression or activity of the enzyme CoA synthase, which converts 3’-dephospho-CoA to CoA in mammalian cells [54]. Further studies are needed to determine at which level this pathway may be perturbed in autoreactive T cells.

Based on these unexpected observations, we investigated the effect of the low molecular weight thiol PTTH, a CoA precursor [10, 43, 60], on autoreactive T cell metabolism and pathogenic potential. CoA is an indispensable metabolite for mammalian cells, involved in more than 100 different biochemical reactions, and about 4% of all known enzymes use it as a cofactor [27]. Thus, we hypothesized that CoA replenishment would alter encephalitogenic T cell metabolic profile and, potentially, their pro-inflammatory and pathogenic activity. Our results confirmed that CoA fueling with PTTH restores CoA synthesis and reshapes the metabolic profile of proliferating autoreactive T cells. Interestingly, the amount of free CoA was found to be reduced at 6–12 h post-treatment. Apart from its rapid utilization by the cells, this reduction might also be caused by feedback mechanisms limiting CoA synthesis when its levels are too high. CoA was indeed suggested to inhibit the activity of several enzymes in its synthesis pathway [28]. More detailed analyses are however necessary to finely assess the kinetics of CoA accumulation and utilization by T cells upon PTTH treatment. Importantly, phosphoproteomics analysis also revealed that PTTH can affect crucial immune processes associated with the functionality and the pathogenicity of encephalitogenic T cells, such as cell activation and proliferation, intravascular T cell adhesion and migration into the CNS. We experimentally confirmed these observations showing that PTTH indeed inhibited encephalitogenic T cell proliferative capacity and pro-inflammatory cytokine production following antigenic stimulation, as well as their integrin-dependent adhesion in vitro and in vivo. This is particularly relevant for MS and other autoimmune and inflammatory disease, where integrin-dependent pathogenic mechanisms are currently a major target for immunotherapy [55]. Strikingly, we didn’t observe a significant effect of PTTH on pan-TCR (CD3/CD28)-activated murine CD4^+^ T cells. These results were unexpected, but may underline differential dynamics of T cell activation. Pan-TCR activation is generally considered stronger than antigen-specific T cell activation, and we used optimal concentrations of anti-CD3 and anti-CD28 antibodies to induce robust T cell proliferation and activation. Lower CD3/CD28 concentration should be titrated to assess whether PTTH may indeed impact T cell functions under sub-optimal pan-TCR stimulation. Of note, we didn’t assess the metabolic profile of CD3/CD28-activated T cells, and an alternative possibility may be differential engagement of the CoA synthesis pathway in T cells upon antigenic *vs* pan-TCR activation. Interestingly, previous work revealed that the metabolic remodeling induced by CD3/CD28 stimulation may not always match the one induced by antigen-specific T cell activation [22]. Future studies evaluating the CoA synthesis pathway upon pan-TCR stimulation are thus warranted to clarify this point.

Considering the metabolomics data showing a significant impact of PTTH treatment on the lipid content of encephalitogenic T cells, we speculate that the immunomodulatory effect of PTTH may be caused, at least in part, by its ability to alter plasma membrane composition and lipid raft formation. Activation of T cells via the TCR is dependent on its localization in cholesterol-rich membrane lipid rafts, which are essential for transmembrane signaling [53, 61]. Moreover, also integrin-dependent signaling is crucial for TCR-mediated T cell activation and leukocyte adhesiveness, and relies on the co-localization of integrins with intracellular signaling hubs in membrane raft structures [29, 53]. Interestingly, a previous work showed that PTTH could inhibit chemokine-dependent adhesion of naïve T cells by altering lipid raft formation on their surface [58], confirming that PTTH may impact T cell activation and functionality by altering membrane lipid composition and lipid raft formation.

In agreement with the significant reduction of encephalitogenic T cell inflammatory profile in vitro, we showed that treatment with PTTH during the pre-clinical phase inhibited the development of both RR-EAE and chronic EAE, suggesting that CoA fueling interferes with immune processes essential for disease onset. This inhibition was associated with a profound immunomodulatory effect of PTTH in EAE mice. These results support a recent study showing that vitamin B5 (pantothenic acid) administration before disease onset limits EAE development in C57Bl mice [14]. Chen and colleagues suggest that CoA dampens the induction of Th17 by targeting the glycolytic enzyme pyruvate kinase M2 (PKM2), which is essential for their development and pro-inflammatory activity [3, 14, 18]. This partially agrees with our data, showing reduced IL-17A production by both murine encephalitogenic T cells and T cells from MS patients, even though we observed a more generalized immunomodulatory effect of PTTH in vitro and in vivo. Noteworthy, we also found that therapeutic administration of PTTH in mice with established EAE reduced disease severity, suggesting that PTTH may interfere with disease-associated processes other than the initial T cell activation in the pre-clinical phase. Of note, differently from CoA and vitamin B5, PTTH may be particularly impactful in neuroinflammatory diseases. In the body, the PTTH molecule can be broken down to pantetheine, pantothenic acid, cystamine and cysteamine, and cystamine and cysteamine were shown to exert significant neuroprotective activity in animal models of neurological diseases [37]. Interestingly, previous studies found that administration of PTTH had beneficial effects in several animal models of neuroinflammation and neurodegeneration, such as cerebral malaria, pantothenate kinase-associate neurodegeneration, Parkinson’s disease and Alzheimer’s disease [6, 11, 15, 16, 38, 43, 57]. In these models, PTTH was shown to have protective effects due to its antioxidant properties and ability to increase mitochondrial functions in damaged neurons [11, 16, 43]. Notably, our metabolomics data showed that PTTH impacts glutathione metabolism in encephalitogenic T cells (Fig. 1b), suggesting that PTTH treatment may also have an antioxidant effect and may induce neuroprotection in EAE mice. Overall, PTTH may at the same time limit neuroinflammation and promote neuronal fitness in EAE, which may explain its beneficial effect in the therapeutic treatment regime.

CoA supplementation is emerging as a novel strategy to regulate inflammation by promoting or limiting immune cell or tissue cell activation [32]. In particular, the effect of PTTH seems to be context-dependent. A first study suggested an anti-inflammatory effect of PTTH in a mouse model of allergic airway inflammation [1]. However, PTTH was recently suggested to promote anti-tumor immunity in mice [31]. This latter work supports an earlier report from St Paul and coworkers showing that the CoA synthesis pathway is enriched in CD8^+^ Tc22 cells, and that fueling T these cells with vitamin B5 boosts their IL-22 production and anti-tumor potential [56]. Thus, depending on the disease and the cellular player analyzed, CoA and its synthesis pathway may play different roles in immunity. At this regard, a crucial part of our work is represented by results demonstrating that treatment with PTTH reduces the pro-inflammatory potential of human Th1 and Th17 cells, as well as T cells from MS patients. Of note, we observed the same trend of inhibition in all the disease forms analyzed (CIS, RRMS, and PMS - data not shown). However, considering the low patient number, the effect of PTTH on T cells from different disease forms needs to be confirmed in larger patient cohorts. Our data thus confirm the immunomodulatory activity of PTTH not only in the mouse system, but also on human T cells, which was never reported before. Notably, these results integrate a recent study showing that ex-vivo treatment with CoA reduced the production of several pro- and anti-inflammatory cytokines by PBMCs from both healthy individuals and MS patients [8], further confirming the immunomodulatory potential of CoA fueling in immune cells. It is interesting to highlight how results in CD3/CD28-activated human CD4^+^ T cells and PBMCs differ from those obtained with resting murine CD4^+^ T cells, where PTTH had no effect on cytokine production upon pan-TCR activation. In this case, the most likely explanation is the higher tolerance of human T cells to the treatment with PTTH, compared to murine T cells. Indeed, the highest, non-toxic PTTH concentration we could use on resting murine T cells (either non-activated encephalitogenic T cell lines or resting CD4^+^ T cells) was 0.5 mM, while human naïve T cells and resting PBMCs were treated with PTTH 1.0 mM with no toxic effect observed. Moreover, while PTTH could not be left in the medium during the 3-day culture of murine T cells because of its toxicity, human T cells were cultured for 5 days in the presence of PTTH. Overall, human T cells seem to be more tolerant to the effect of CoA fueling with PTTH. The reason for this is unclear, but it may underscore metabolic and/or functional differences between human and mouse T cells.

## Conclusions

Overall, our work, together with previous reports showing its neuroprotective and antioxidant activity, suggest that PTTH may represent a novel therapeutic option for the treatment of neuroinflammation. PTTH exerts a significant immunomodulatory effect on T cells, reducing their overall pro-inflammatory potential. However, our results also suggest that it does not promote the generation of Tregs in human T cell cultures, which is a major goal of autoimmune disease therapies [52]. We thus propose that PTTH supplementation may serve as an adjuvant treatment in MS and other human neuroinflammatory and autoimmune disorders in combination with disease-modifying drugs, in order to boost their overall efficacy. Importantly, PTTH has been used for years as a lipid-lowering drug in humans, with very minor side effects reported [57]. Its translation to the clinical practice in patients suffering with such pathologies may thus be rapid.

## Supplementary Information

**Additional file 1. Supplementary Data 1.** Raw data and results of the metabolomics analysis.

**Additional file 2. Supplementary Data 2.** Raw data and results of the phosphoproteomics analysis. 

**Additional file 3: Figure S1.** Metabolomics analysis of resting T cells and actively-proliferating encephalitogenic T cells before and after PTTH treatment. **a** PCA scores plot showing each samples’ metabolome as dot where nearness represents similarity. Clear group separation is visible between all four groups, with the strongest difference resulting from T cell type along PC1 followed by the impact from PTTH treatment along PC2. **b** PLS-DA scores plot confirms that group separation is significant with ROC analysis of each group against all other groups, all resulting in AUC > 0.95 and Wilcoxon **p < 0.01, and ***p < 0.001. Ellipses mark the groups 95% confidence interval. **c** Dumbbell plot of metabolites without any significant change in any of the three comparisons of interest. The plot shows the strength of metabolic changes along the x-axis, the metabolite name and class on the y-axis with thin lines connecting the same metabolite. The significance is encoded in shapes (*p *≥ 0.05).

**Additional file 4: Figure S2.** PTTH fuels CoA in actively-proliferating encephalitogenic T cells. **a** PTTH structure. **b** Left: quantification of CoA levels in untreated (CTRL) and PTTH-treated encephalitogenic T cells (PTTH 1.0 mM). Right: ratio between CoA concentrations in PTTH-treated and CTRL cells. Data are the mean ± SD of N = 4 independent experiments. **p < 0.01 by 2-way Anova with Šídák’s test for multiple comparisons (left graph) and *p < 0.05 by Friedman test with Dunn’s test for multiple comparisons (right graph).

**Additional file 5: Figure S3.** MAPK network identified by network modularization with MCODE.

**Additional file 6: Figure S4.** Effect of PTTH on CD3/CD28-activated murine T cells. Murine CD4^+^ resting T cells were incubated for 8 h with vehicle (CTRL) or PTTH 0.5 mM, washed, and polarized in vitro towards Th0, Th1, Th2 and Th17 cells. **a** Cell proliferation was determined as CTV dilution after 3 days of culture. **b-e** Cytokine production was evaluated in Th0 (**b**), Th1 (**c**), Th2 (**d**) and Th17 (**e**) cells by intracellular cytokine staining. In all panels, data are the mean ± SD of N = 5 independent mice.

**Additional file 7: Figure S5.** PTTH does not affect human Th1 and Th17 cell proliferation and Treg development. **a** Human Th1 and Th17 cells were polarized in vitro from CD4^+^ naïve T cells in the presence of vehicle (CTRL) or PTTH 1.0 mM. Cell proliferation was determined as CTV dilution after 5 days of culture. Data are the mean ± SD of N = 10 independent donors. **b** Human CD4^+^ naïve T cells were polarized in vitro to Th1, Th17, and induced Tregs (iTregs). The amount of CD25^+^Foxp3^+^ was quantified at the end of the culture on the CD127^−^ population. Data are the mean ± SD of N = 10 independent donors.

## Data Availability

The data supporting the findings of this study are available in the main manuscript and in the supplementary materials. Raw data are available upon request from the corresponding authors.

## Abbreviations

EAE: Experimental autoimmune encephalomyelitis
MS: Multiple sclerosis
CoA: Coenzyme A
PTTH: Pantethine
Th0: T helper 0
Th1: T helper 1
Th2: T helper 2
Th17: T helper 17
Tregs: Regulatory T cells
TCA: Tricarboxylic acid
CNS: Central nervous system
PLP: Proteolipid protein
APC: Antigen-presenting cell
MVA and UVA: Multivariate analysis and univariate analysis
PCA: Principal component analysis
PLS-DA: Partial least-squares discriminant analysis
MAPK: Mitogen-activated protein kinase
ICAM-1: Intercellular cell adhesion molecule-1
VCAM-1: Vascular cell adhesion molecule-1
LPS: Lipopolysaccharide
RR-EAE: Relapsing–remitting EAE
MOG: Myelin oligodendrocyte glycoprotein
PBMCs: Peripheral blood mononuclear cells
IL: Interleukin
CTV: CellTraceViolet
PMA: Phorbol-12-myristate-13-acetate
IFN-γ: Interferon-gamma
GM-CSF: Granulocyte–macrophage colony-stimulating factor
TNF-α: Tumor necrosis factor-alpha
TCR: T-cell receptor
