# Supplementary figures and images for: Coenzyme A fueling with pantethine limits autoreactive T cell pathogenicity in experimental neuroinflammation
Source: J Neuroinflammation. 2024 Nov 5;21:287. doi: 10.1186/s12974-024-03270-w (PMC11536535; doi:10.1186/s12974-024-03270-w)

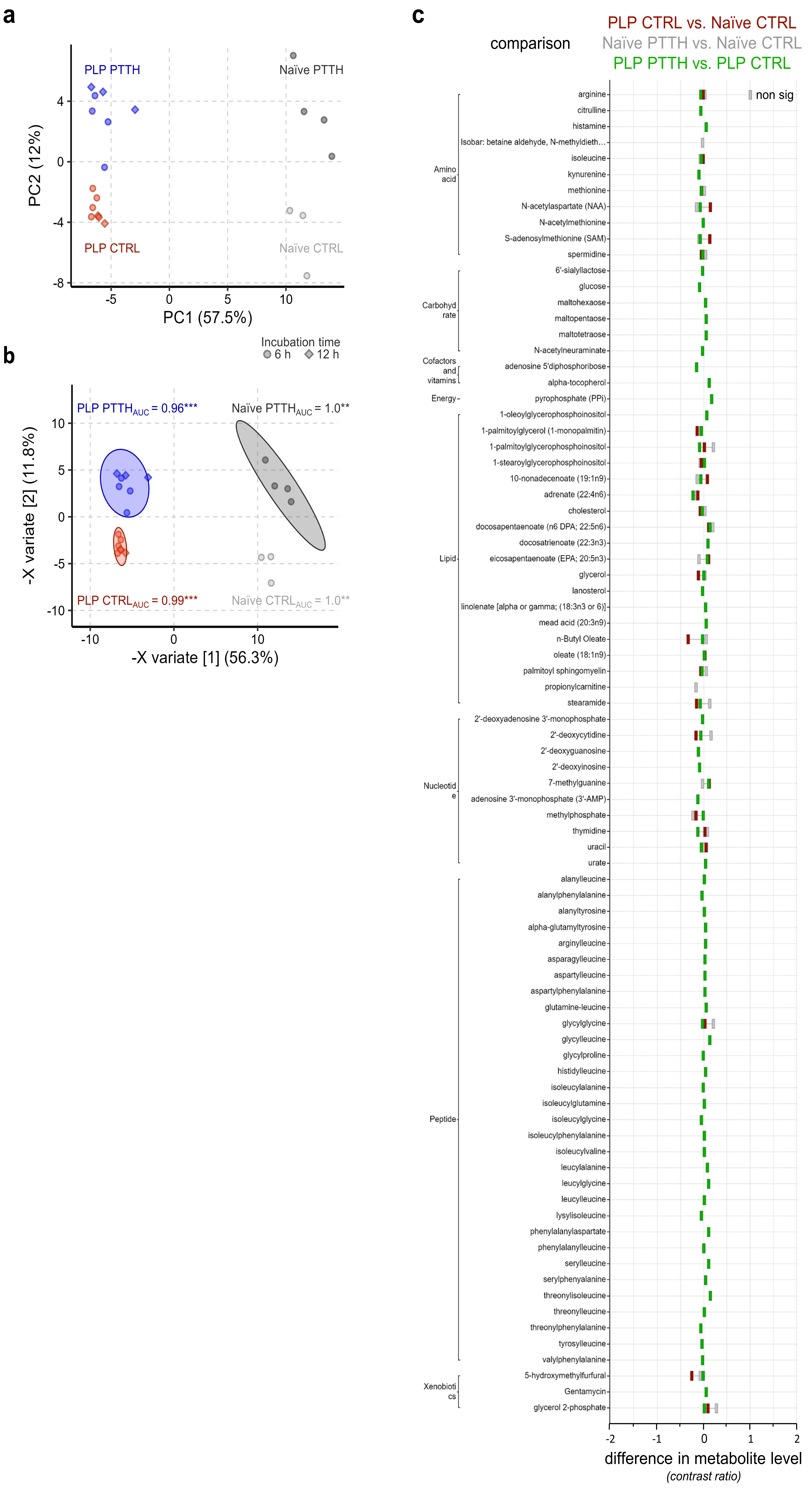

Supplement: Supplementary file 3 — Additional file 3: Figure S1. Metabolomics analysis of resting T cells and actively-proliferating encephalitogenic T cells before and after PTTH treatment. a PCA scores plot showing each samples’ metabolome as dot where nearness represents similarity. Clear group separation is visible between all four groups, with the strongest difference resulting from T cell type along PC1 followed by the impact from PTTH treatment along PC2. b PLS-DA scores plot confirms that group separation is significant with ROC analysis of each group against all other groups, all resulting in AUC > 0.95 and Wilcoxon **p < 0.01, and ***p < 0.001. Ellipses mark the groups 95% confidence interval. c Dumbbell plot of metabolites without any significant change in any of the three comparisons of interest. The plot shows the strength of metabolic changes along the x-axis, the metabolite name and class on the y-axis with thin lines connecting the same metabolite. The significance is encoded in shapes (p ≥ 0.05). [file 12974_2024_3270_MOESM3_ESM.png]

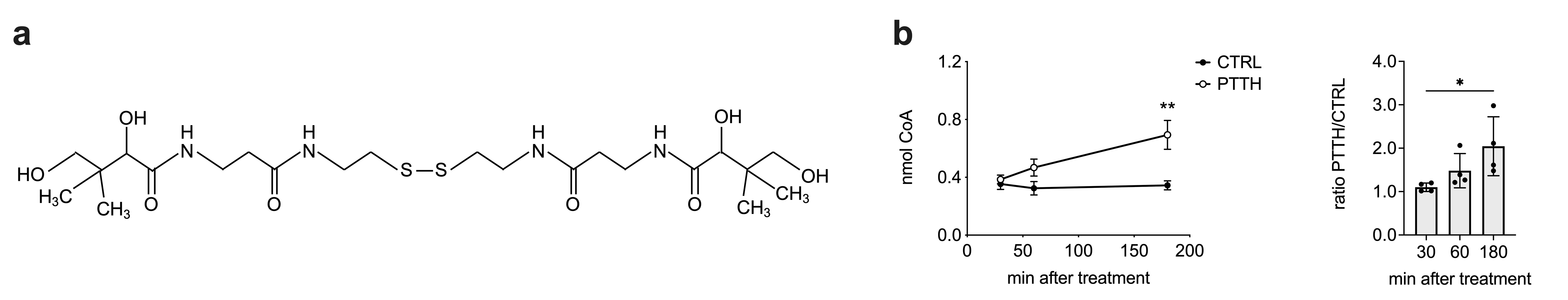

Supplement: Supplementary file 4 — Additional file 4: Figure S2. PTTH fuels CoA in actively-proliferating encephalitogenic T cells. a PTTH structure. b Left: quantification of CoA levels in untreated (CTRL) and PTTH-treated encephalitogenic T cells (PTTH 1.0 mM). Right: ratio between CoA concentrations in PTTH-treated and CTRL cells. Data are the mean ± SD of N = 4 independent experiments. **p < 0.01 by 2-way Anova with Šídák’s test for multiple comparisons (left graph) and *p < 0.05 by Friedman test with Dunn’s test for multiple comparisons (right graph). [file 12974_2024_3270_MOESM4_ESM.tiff]

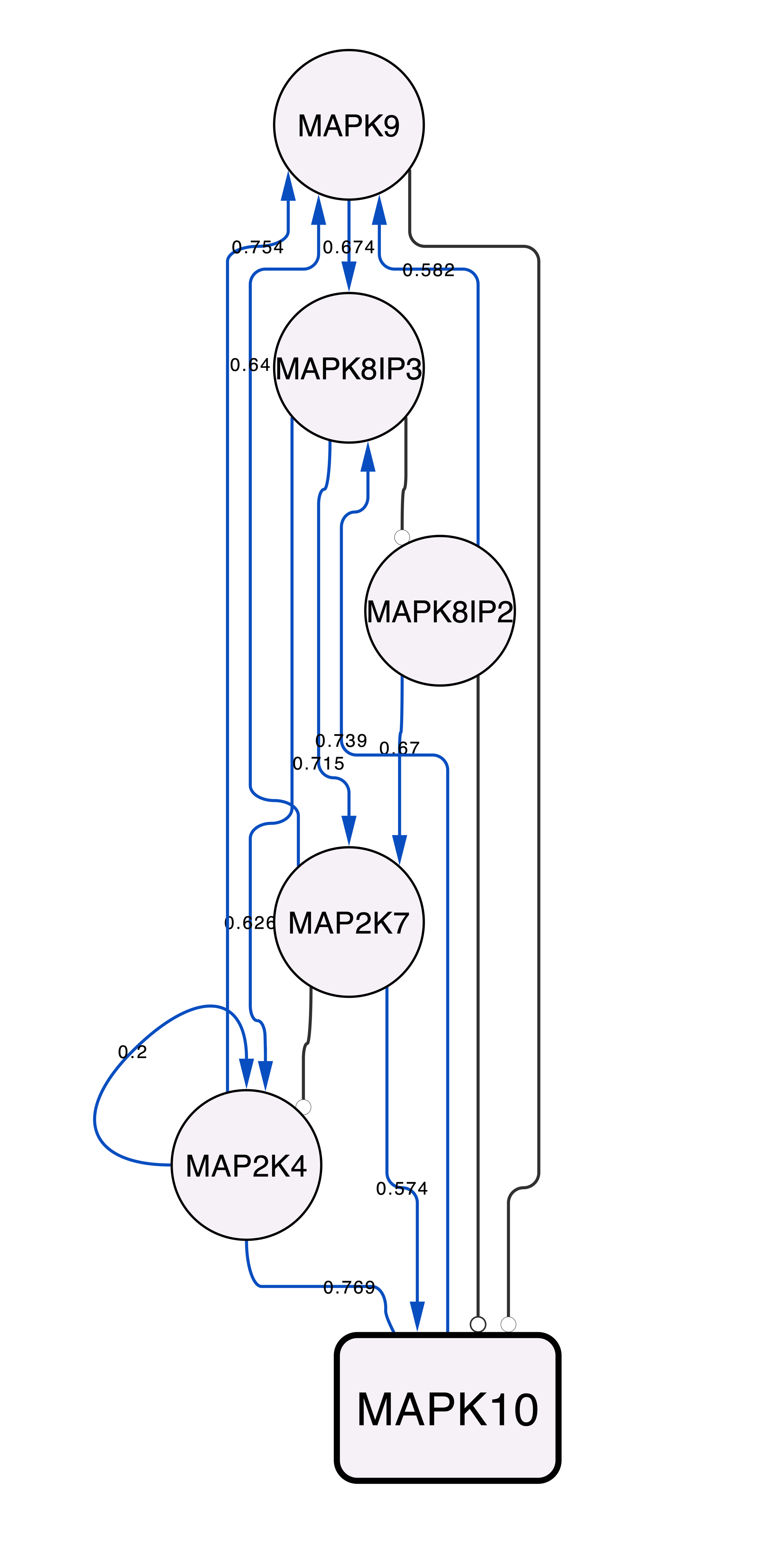

Supplement: Supplementary file 5 — Additional file 5: Figure S3. MAPK network identified by network modularization with MCODE. [file 12974_2024_3270_MOESM5_ESM.tiff]

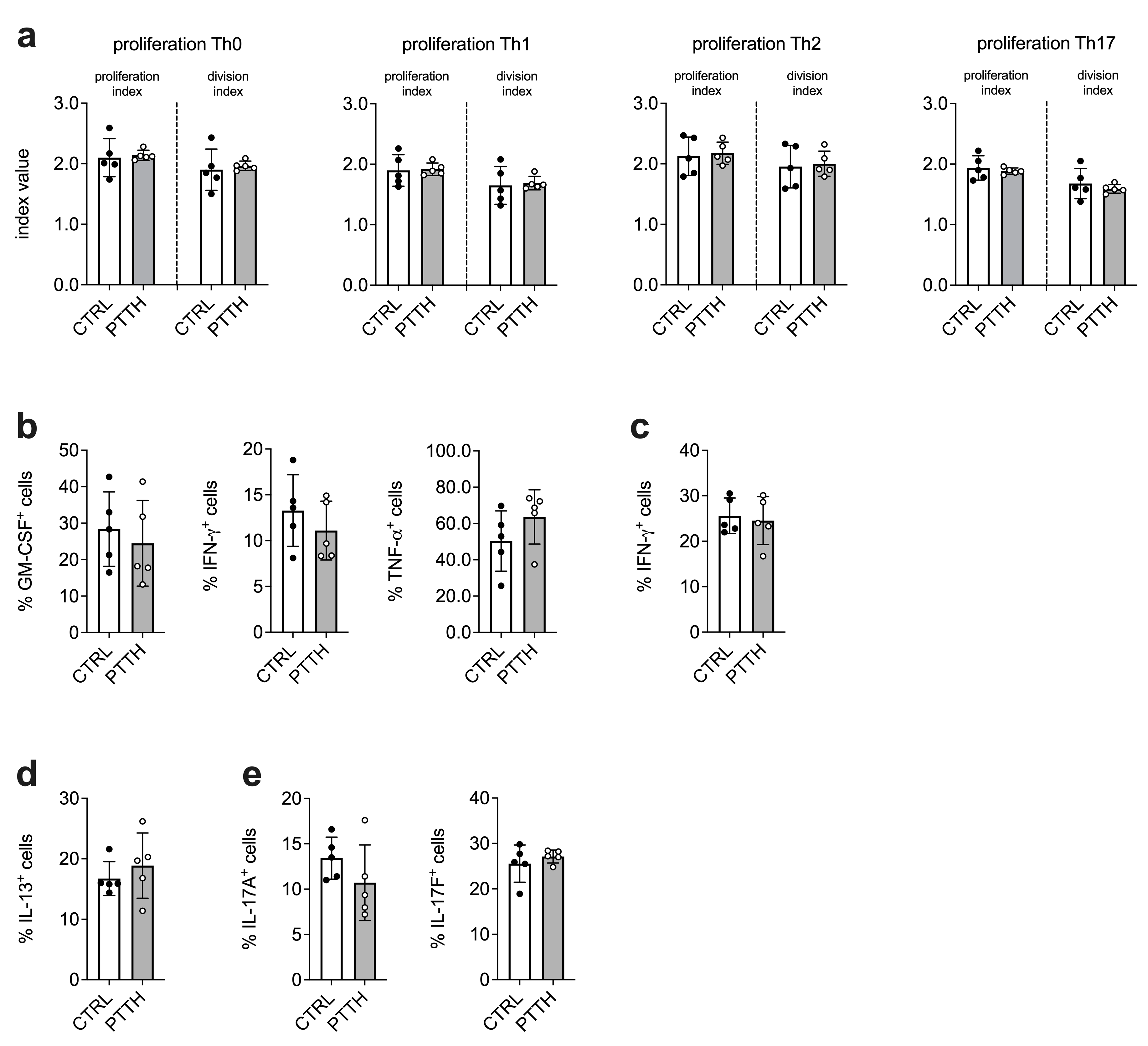

Supplement: Supplementary file 6 — Additional file 6: Figure S4. Effect of PTTH on CD3/CD28-activated murine T cells. Murine CD4+ resting T cells were incubated for 8 h with vehicle (CTRL) or PTTH 0.5 mM, washed, and polarized in vitro towards Th0, Th1, Th2 and Th17 cells. a Cell proliferation was determined as CTV dilution after 3 days of culture. b-e Cytokine production was evaluated in Th0 (b), Th1 (c), Th2 (d) and Th17 (e) cells by intracellular cytokine staining. In all panels, data are the mean ± SD of N = 5 independent mice. [file 12974_2024_3270_MOESM6_ESM.tiff]

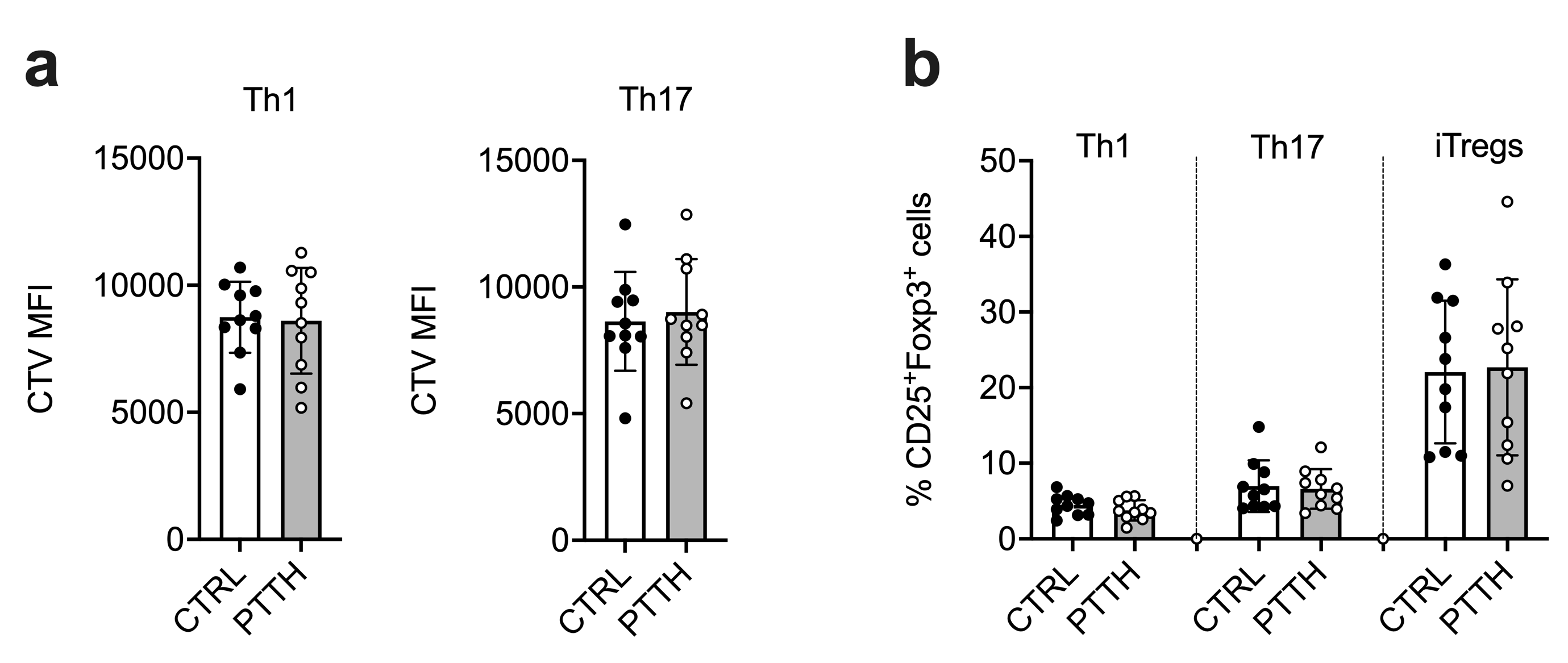

Supplement: Supplementary file 7 — Additional file 7: Figure S5. PTTH does not affect human Th1 and Th17 cell proliferation and Treg development. a Human Th1 and Th17 cells were polarized in vitro from CD4+ naïve T cells in the presence of vehicle (CTRL) or PTTH 1.0 mM. Cell proliferation was determined as CTV dilution after 5 days of culture. Data are the mean ± SD of N = 10 independent donors. b Human CD4+ naïve T cells were polarized in vitro to Th1, Th17, and induced Tregs (iTregs). The amount of CD25+Foxp3+ was quantified at the end of the culture on the CD127− population. Data are the mean ± SD of N = 10 independent donors. [file 12974_2024_3270_MOESM7_ESM.tiff]
